# Recent Advances in Lemongrass Essential Oil: Food Safety, Preservation, and Bioactivity in Food Systems

**DOI:** 10.1111/1541-4337.70350

**Published:** 2025-12-05

**Authors:** Ahmad Rabbani, Ayman Khaliq, Priti Mudgil, Sajid Maqsood, Akmal Nazir

**Affiliations:** ^1^ Department of Food Science, College of Agriculture and Veterinary Medicine United Arab Emirates University Al Ain United Arab Emirates

**Keywords:** antimicrobial activity, antioxidant activity, coatings, edible films, food preservation, lemongrass essential oil

## Abstract

Lemongrass essential oil (LGEO) has gained recognition as a natural preservative in food systems due to its distinctive phytochemical composition and multifunctional bioactivities. Dominated by citral and complemented by terpenes and phenolic compounds, LGEO demonstrates potent antibacterial, antifungal, antiviral, antibiofilm, and antioxidant effects, collectively enhancing food safety and quality. These properties align with growing consumer demand for natural and sustainable additives, offering an attractive alternative to synthetic preservatives. This review consolidates current knowledge of LGEO extraction methods, their chemical composition, mechanisms of action, and practical integration into food applications. Particular emphasis is placed on emerging technologies such as edible films and coatings, active and intelligent packaging, emulsion‐based systems, and micro‐ and nanoencapsulation, which improve stability, mitigate volatility, and enable controlled release. We also summarize current knowledge on its toxicological profile and highlight regulatory considerations for safe use in foods. This review provides a comprehensive perspective on LGEO's potential in modern food preservation strategies by bridging insights from chemistry, bioactivity, and food technology.

## 1 Introduction

Essential oils (EOs) derived from aromatic plants have long been recognized for their bioactive properties and are increasingly being explored as natural alternatives to synthetic preservatives in food systems (Al‐Maqtari et al. 2021). EOs have been extracted from numerous aromatic herbs, including oregano, thyme, basil, clove, and rosemary, each with varying degrees of biological activity and market relevance. Among these, lemongrass essential oil (LGEO), obtained mainly from *Cymbopogon citratus* and *Cymbopogon flexuosus*, has emerged as a high‐potential option due to its superior EO yield (1.5%–2.5% v/w fresh biomass), which significantly surpasses other EOs such as basil (0.3%–1.1%) or thyme (0.5%–1.3%) under standard cultivation conditions (Bajrang et al. 2025; Kumoro et al. 2021). Beyond biomass yield, LGEO is distinguished by a unique phytochemical profile dominated by citral isomers, which constitute up to 75%–85% of its composition. This confers broad‐spectrum antimicrobial, antioxidant, and antifungal activities, more pronounced and consistent than many other EO plants (Rhimi et al. 2022; Faheem et al. 2022).

**FIGURE 1:**
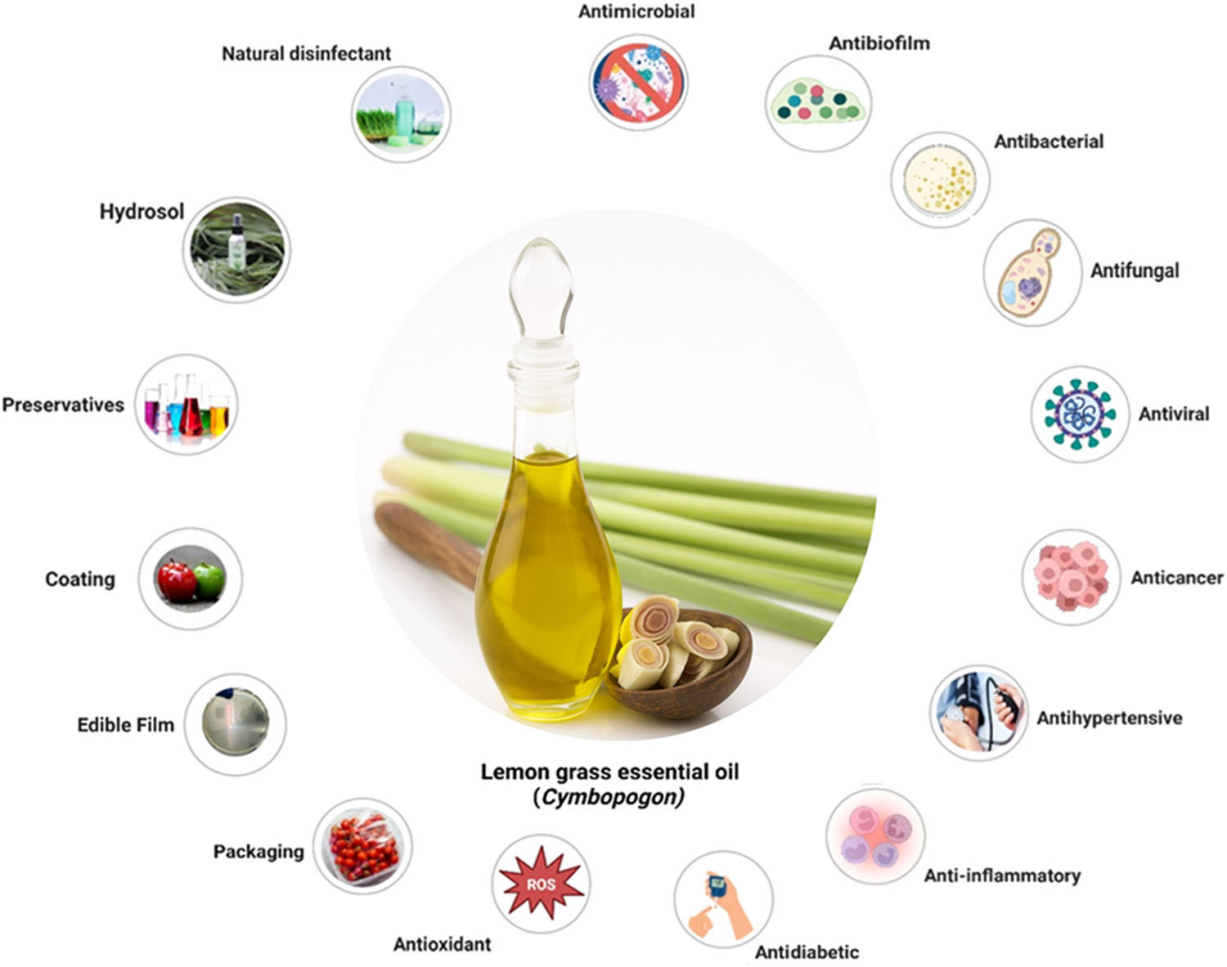
Overview of the major applications of lemongrass essential oil (*Cymbopogon* spp.). Created in BioRender (https://BioRender.com/74gwlo4).

Traditionally employed in folk medicine, aromatherapy, and the cosmetic industry, LGEO is now positioned at the intersection of food safety, quality enhancement, and sustainability (Ashaq et al. 2024). The global shift toward “clean‐label” products has driven strong consumer demand for natural additives that can replace or reduce synthetic chemicals in food preservation (Kumar et al. 2025). Safety and regulatory approval further strengthen the case for LGEO. The United States Food and Drug Administration (FDA) recognizes it as “generally recognized as safe” (GRAS). LGEO demonstrates broad‐spectrum antimicrobial activity against bacteria, fungi, and viruses, as well as antibiofilm activity, particularly relevant for controlling persistent foodborne pathogens (Faheem et al. 2022; Ashaq et al. 2024). Comparative studies have shown LGEO's superior efficacy over clove, basil, and rosemary oils in food preservation, biofilm inhibition, and emulsion‐based delivery systems (Kaur et al. 2024; Selim et al. 2025). It has shown effective inhibition against *Listeria monocytogenes*, *Salmonella enterica*, and *Escherichia coli*, outperforming rosemary, basil, and thyme oils (Torres Neto et al. 2022; Vidaković Knežević et al. 2023). Figure 1 represents the major applications of LGEO.

In parallel, its antioxidant potential, attributed primarily to citral and supported by minor constituents such as limonene, geraniol, and flavonoids, contributes to delaying lipid oxidation and preventing quality deterioration in perishable foods (do Nascimento et al. 2023; Putra et al. 2025; Singh et al. 2024). These combined properties make LGEO a versatile agent for enhancing both food safety and shelf life. Beyond its inherent bioactivity, technological innovations have expanded the possibilities for LGEO utilization in modern food systems. Its incorporation into edible films, coatings, and active or intelligent packaging has demonstrated efficacy in extending the postharvest life of fruits, vegetables, and bakery products (Manjunatha et al. 2023; Cruz et al. 2023). Similarly, its formulation into emulsions, nanoemulsions, and encapsulated systems improves stability, overcomes volatility, and enables controlled release (Salvia‐Trujillo et al. 2014). These delivery strategies not only enhance functional performance but also mitigate sensory challenges associated with LGEO's strong lemon‐like flavor, thereby broadening its acceptability across diverse food matrices (Adhikary et al. 2023; Ashaq et al. 2024).

Research activity on LGEO has expanded considerably in recent years, with more than 400 research articles indexed in Scopus to date. Despite this growing body of primary research, relatively few comprehensive reviews have been published. Among the recent reviews, some have concentrated on the extraction methods of LGEO (e.g., Adhikary et al. 2023; Ashaq et al. 2024), others have focused on its bioactivities (Mukarram et al. 2021; Silva et al. 2022; Faheem et al. 2022; Ashaq et al. 2024), while Moreira da Silva et al. (2024) emphasized encapsulation approaches. However, to the best of our knowledge, no review has comprehensively synthesized LGEO's role specifically within the food system. By briefly addressing extraction alongside detailed discussions on chemical composition, mechanisms of action, and applications in films, coatings, packaging, emulsions, and encapsulation, this review uniquely highlights the role of LGEO at the intersection of food safety and food preservation strategies. Moreover, safety, toxicological evidence, and regulatory perspectives are also addressed, followed by an exploration of limitations and future directions. By consolidating insights from recent studies, this review aims to highlight LGEO's potential as a sustainable, multifunctional natural preservative and to identify research priorities to fully realize its role in advancing food safety and quality.

## 2 Extraction and Typical Chemical Composition

LGEO extraction can be achieved through a range of conventional and modern techniques, each differing in efficiency, scalability, and impact on oil quality. Steam distillation remains the most widely used and preferred method due to its simplicity, cost‐effectiveness, and industrial scalability, and it continues to serve as the reference technique despite the availability of more advanced approaches (Balti et al. 2018; Gogoi et al. 2026; Sayal et al. 2025). Hydrodistillation, often performed with a Clevenger apparatus, follows similar principles but involves direct boiling of plant material in water, with specific water‐to‐plant ratios (e.g., 1:8 or 12:1) reported to influence both yield and composition (Bag et al. 2022; Chepyala et al. 2025; Vargas et al. 2010). Solvent extraction, typically employing *n*‐hexane, enables recovery of a broader profile of volatile and semivolatile compounds but is limited by longer processing times, higher energy demands, and the risk of solvent residues in the oil (Ashaq et al. 2024; Balti et al. 2018; Moncada et al. 2014). To overcome these drawbacks, several modern and green extraction techniques have been developed. Supercritical fluid extraction (SFE) with CO_2_ offers high selectivity and efficiency while minimizing solvent use, making it an environmentally favorable option (Ashaq et al. 2024; Moncada et al. 2014). Similarly, ultrasound‐assisted extraction (UAE) and microwave‐assisted techniques (MAE/MAHD) have been shown to significantly reduce extraction time and energy consumption while preserving heat‐sensitive compounds, with solvent‐free microwave extraction (SFME) providing an especially sustainable alternative (Ashaq et al. 2024; Chouhan et al. 2023; Sayal et al. 2025). Maceration, involving prolonged soaking of plant material in solvents at ambient conditions, is less favored for LGEO due to inefficiency and the potential degradation of volatile constituents. For a more detailed comparative evaluation of these conventional and modern extraction strategies, readers are referred to the comprehensive review by Ashaq et al. (2024).

**FIGURE 2:**
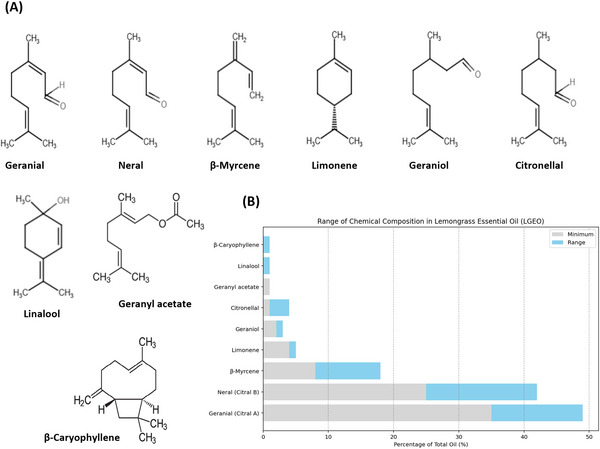
Principal chemical constituents of lemongrass essential oil (LGEO) with their (A) molecular structures and (B) typical compositional ranges.

LGEO is characterized by a complex mixture of chemical constituents, with oxygenated monoterpenes as the dominant group, typically representing 78%–81% of the oil's composition. Monoterpene hydrocarbons form a smaller but consistent fraction, generally around 8%–10%, while sesquiterpenes and sesquiterpene hydrocarbons occur in minor amounts, usually less than 4% (Hadjilouka et al. 2017; Tyagi and Malik 2010). This predominance of oxygenated monoterpenes is primarily attributed to citral, a mixture of two isomeric monoterpene aldehydes: geranial (*trans*‐citral) and neral (*cis*‐citral). Citral is responsible for the oil's strong lemon‐like aroma and bioactivity, which imparts the characteristic lemon aroma and supports many of LGEO's functional properties in food systems. It is the dominant component in most lemongrass oils, often comprising around 65%–85% of the total oil. Among the citral isomers, geranial is usually present in a slightly higher proportion than neral (geranial often accounts for about 40%–62% of the oil, compared to 5%–38% for neral; Gao et al. 2020; Hadjilouka et al. 2017; Tazi et al. 2024). Beyond citral, LGEO contains several secondary terpenes and terpenoids, notably β‐myrcene, limonene, and geraniol, which contribute to its fragrance and enhance bioactivity through synergistic effects (Almeida et al. 2024; Hadjilouka et al. 2017; Mishra et al. 2021). Among these, β‐myrcene is often the second‐most abundant constituent after citral and is recognized for its analgesic and antibacterial properties (Kiani et al. 2022). Other compounds, such as citronellal, geranyl acetate, linalool, and β‐caryophyllene, are typically detected in trace amounts, each contributing approximately 1% or less to the overall oil composition (Mishra et al. 2021; Yogendra et al. 2024). Figure 2 shows the principal chemical constituents of LGEO and their typical concentrations as reported in the literature.

A complex interplay of genetic, environmental, and technological factors influences the chemical composition of LGEO. Species and chemotype variations (e.g., *C. citratus* vs. *C. flexuosus*) are primary determinants of the relative abundance of major constituents such as geranial and neral, reflecting inherent genetic differences (Mwithiga et al. 2022). Geographical origin and agroecological conditions also exert a pronounced effect, with factors such as altitude, soil composition, and climatic zone influencing both yield and chemical profile; notably, higher citral levels have been associated with basic soils and lower altitudes (Freitas and Cattelan 2018; Pandey et al. 2020). Growing conditions, including rainfall, temperature, sunlight, and humidity, modulate the biosynthesis and accumulation of EO constituents, while agronomic practices such as irrigation management, nutrient supplementation, and photoperiod control contribute to seasonal and interharvest variability (Jaouad et al. 2025; Mwithiga et al. 2022; Pandey et al. 2020). Harvest timing further impacts both oil quality and complexity, with plant age and seasonal factors altering citral proportions and the diversity of detectable compounds; for example, significant compositional shifts have been observed in plants harvested at 4.5, 6.5, and 7.5 months (Hadjilouka et al. 2017). Additionally, extraction and pretreatment methods significantly impact the final composition. Differences among steam distillation, hydrodistillation, and supercritical CO_2_ extraction have been reported, while pretreatment steps such as drying (sun, shade, or oven) and the use of fresh versus dried material can lead to citral variations of up to 8% (Jaouad et al. 2025; Pourmortazavi and Hajimirsadeghi 2007).

## 3 Mechanisms of Action in Food Matrices

LGEO exhibits a wide spectrum of bioactive properties, directly linked to its diverse phytochemical composition, with citral isomers and associated terpenes playing central roles. These properties are particularly relevant in the context of food systems, where LGEO has been investigated for its ability to inhibit pathogenic and spoilage microorganisms, suppress biofilm formation, and mitigate oxidative deterioration. The following subsections provide a detailed synthesis of LGEO's antimicrobial and antioxidant activities, highlighting mechanistic insights, experimental evidence, and applications in food preservation.

### 3.1 Antibacterial Activity

LGEO exhibits pronounced antibacterial activity against a broad spectrum of foodborne and opportunistic pathogens, including both Gram‐negative and Gram‐positive bacteria (Table 1). Sensitive Gram‐negative strains include *Serratia marcescens*, *E. coli*, *Pseudomonas aeruginosa*, *Chromobacterium violaceum* (Du et al. 2024; Gao et al. 2020), *Salmonella typhimurium* (Rabbani et al. 2025), *Proteus vulgaris* (Sheriff Maqbul et al. 2022), *Enterobacter aerogenes* (Mostoles et al. 2017), and *Salmonella paratyphi* (Kayiran et al. 2025). Among Gram‐positive organisms, *Staphylococcus aureus*, *Staphylococcus albus* (Gao et al. 2020), *Bacillus subtilis* (Hirsch et al. 2024), *Bacillus cereus*, *L. monocytogenes* (Han Lyn and Nur Hanan 2020), *Lactobacillus buchneri*, *L. hilgardii* (Del Valle et al. 2025), and *Enterococcus faecalis* (Khaliq et al. 2024) have all been reported as susceptible. This broad inhibitory range indicates that LGEO targets both simple and more structurally complex bacterial cell envelopes, reflecting its potential as a versatile antimicrobial in food systems.

**TABLE 1:** Summary of selected studies on the antimicrobial activity of lemongrass essential oil.

Microorganism targeted	Result (MIC/MBC/ZOI)	Outcome	References
*Vibrio parahaemolyticus* (multidrug‐resistant)	Minimum biofilm inhibitory concentration (MBIC): 0.25% LGEO	LGEO reduced biofilm formation by 85% in fish samples, preventing bacterial adhesion on food surfaces	Alasgah et al. (2025)
*Staphylococcus aureus*, *E. coli*	*S. aureus*: 18 mm ZOI at 0.4% LGEO, *E. coli*: 14 mm ZOI	LGEO incorporated in chitosan‐PVA nanocomposite films increased antimicrobial effectiveness and prolonged bacterial inhibition for 21 days	Daeialiakbar et al. (2025)
*S. aureus*, *E. coli*	*S. aureus*: MIC: 0.25%, *E. coli*: MIC: 0.4%	LGEO nanoformulations improved stability and antibacterial effects by 3 × compared to free LGEO.	Noorbakhsh et al. (2025)
*Staphylococcus aureus*, *E. coli*	MIC: 20 µg/mL, 40 µg/mL MBC: 30 µg/mL, 40 µg/mL ZOI (mm): 52.5 ± 2, 23.5 ± 1	The vitamin E nanoemulsion significantly enhanced antibacterial efficacy, demonstrating 5.68 × greater inhibition than pure vitamin E, particularly against *Staphylococcus aureus*. However, its effectiveness against *Escherichia coli* was relatively lower, with an inhibition increase of 2.61 ×, indicating stronger antibacterial properties in Gram‐positive bacteria due to nanoencapsulation.	Prakash et al. (2025)
*Salmonella typhimurium* (DT 104)	MIC: 0.09%	LGEO nanoemulsion can inhibit the growth of *Salmonella typhimurium* at different temperatures for a prolonged time.	Rabbani et al. (2025)
*Pseudomonas aeruginosa, Bacillus subtilis, Staphylococcus aureus*	MIC: 0.1%–0.25%, MBC: 0.2%–0.35%, ZOI: 19–24 mm	β‐Citral identified as key antimicrobial compound. Strong inhibition against Gram‐negative bacteria.	Weshahi et al. (2025)
*Listeria monocytogenes, Salmonella Typhimurium*	MIC: 0.3%–0.5%, MBC: 0.6%–0.7%, ZOI: 18–22 mm	Synergistic effects of lemongrass oil with bergamot and lime essential oils increased efficacy.	Lakkana et al. (2024)
*Staphylococcus aureus*, *Escherichia coli*, *Candida albicans*	MIC: 0.2%–0.4%, MBC: 0.3%–0.5%, ZOI: 17–26 mm	A microemulsified hydrogel formulation extended antibacterial activity up to 7 days.	Nguyen et al. (2024)
*Salmonella enterica* *Escherichia coli* *Listeria monocytogenes* *Staphylococcus aureus*	MIC: 0.3%–0.6%, MBC: 0.5%–0.8%, ZOI: 18–21 mm	GC–MS confirmed high levels of citral and limonene, which were responsible for antibacterial activity.	Ariani et al. (2025)
*E. coli*, *Staphylococcus aureus*, *Pseudomonas aeruginosa*, *Candida albicans*, *Listeria monocytogenes*, *Salmonella enterica*	MIC: Ranged from 0.1% to 0.5%, MBC: 0.2%–0.7%, ZOI: 15–24 mm	Lemongrass essential oil demonstrated strong inhibition against Gram‐positive and Gram‐negative bacteria, fungi, and foodborne pathogens. It exhibited high synergistic effects when used with other essential oils. Biofilm reduction was observed in *E. coli* and *S. aureus*, and fungicidal activity was notable against *Candida albicans*.	Mukarram et al. (2022)
*Staphylococcus aureus Staphylococcus epidermidis Escherichia coli*, *Klebsiella pneumoniae*, *Candida albicans*, *Candida parapsilosis*, *Candida tropicalis*	Bacteria: MIC: 0.2%–0.5%, MBC: 0.4%–0.7%, ZOI: 18–26 mm depending on strain.—Fungi: MIC: 0.1%–0.4%, ZOI: 15–22 mm	LGEO showed strong antibacterial effects, even against multidrug‐resistant (MDR) bacteria. The highest inhibition zones were recorded for *S. aureus* (26 mm) and *E. coli* (24 mm). Antifungal tests showed strong inhibition of *Candida albicans* and *C. tropicalis* biofilms.	Soares et al. (2013)
*Aeromonas hydrophila*	MIC: 31 µL/mL Biofilm Reduction: 4.51 log CFU/cm^2^	LGEO demonstrated a significant reduction in biofilm (4.51 log cycles) on stainless steel surfaces. SEM analysis confirmed biofilm disruption and bacterial dispersion after treatment, demonstrating LGEO's potential as a natural disinfectant.	Millezi et al. (2013)
*Aspergillus flavus*	MIC: 500 ppm—MBC: 750 ppm—ZOI: Not directly mentioned	LGEO completely inhibited mycelial growth of *A. flavus* at 750 ppm and suppressed aflatoxin B1 production by 100% at 500 ppm. The *in vivo* fumigation of stored root samples with 500 ppm EO resulted in 92% protection from fungal infestation, making it a potential natural antifungal agent for herbal storage.	Singh et al. (2010)
*Aspergillus flavus, Aspergillus parasiticus, Aspergillus ochraceus, Aspergillus niger, Aspergillus fumigatus*	MIC: 15–118 mg/mL—ZOI: 19.6–46.3 mm	LGEO exhibited strong antifungal properties against *A. niger*. The oil showed dose‐dependent inhibition, with the highest antifungal activity attributed to citral (geranial and neral). *A. flavus* exhibited the highest resistance (MIC: 118 mg/mL). The study supports lemongrass EO as a natural alternative for grain preservation to inhibit mycotoxin‐producing fungi.	Matasyoh et al. (2011)
*Staphylococcus aureus, Staphylococcus epidermidis, Enterococcus faecalis, Mycobacterium smegmatis, Malassezia furfur, Candida albicans*	ZOI: 10.3–30.3 mm. MIC: 0.19% v/v and 0.12% v/v MBC/MFC: 0.12% v/v and 0.19% v/v	LGEO exhibited antibacterial and antifungal activities, with the highest inhibition zones observed against *Mycobacterium smegmatis* (30.3 mm) and *Candida albicans* (28.3 mm). Additionally, *Malassezia furfur* and *C. albicans* were inhibited entirely at 0.12% v/v.	Khaliq et al. (2024)

**FIGURE 3:**
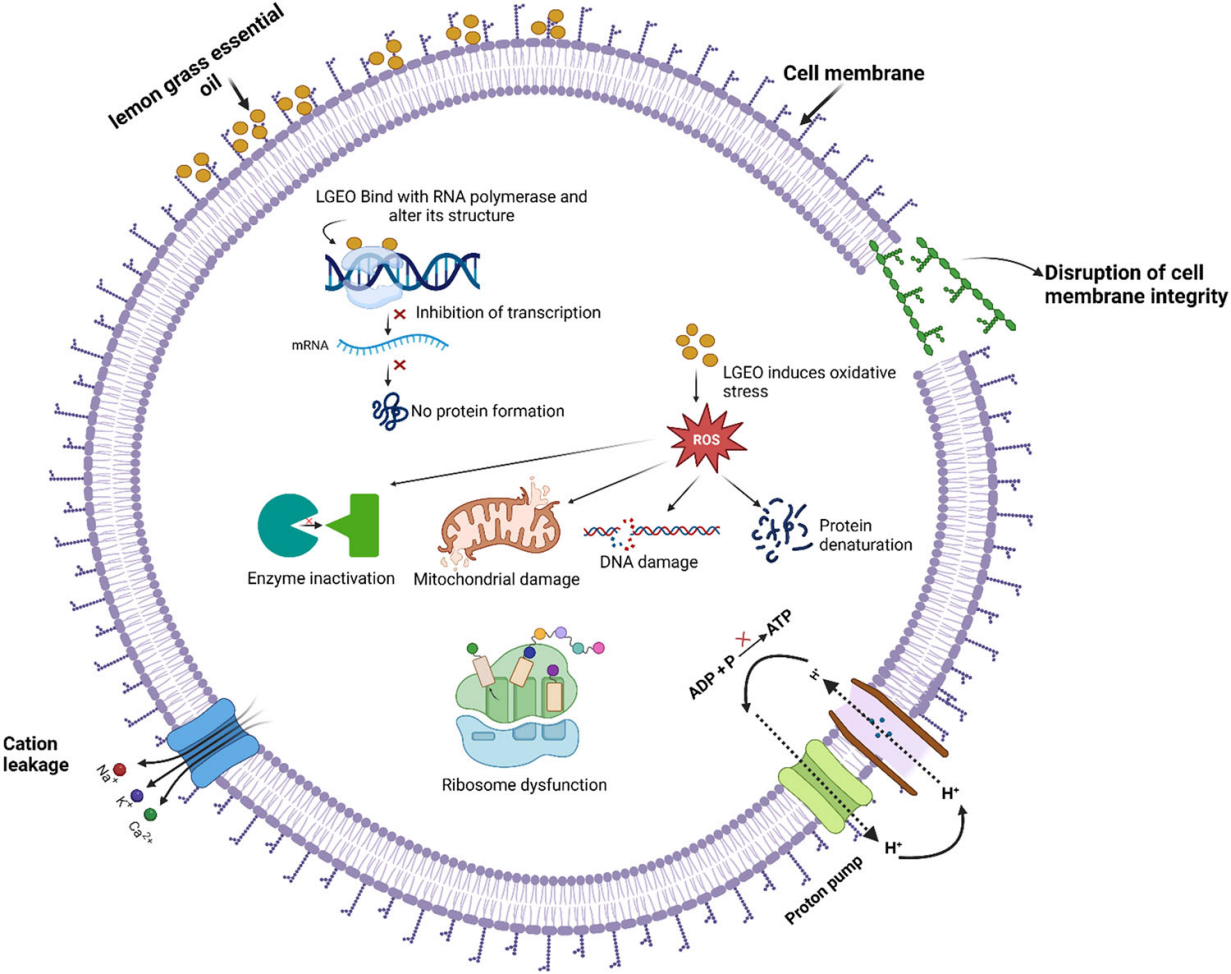
Possible antibacterial mechanisms of lemongrass essential oil. Created in BioRender (https://BioRender.com/57inthz).

The antibacterial effect of LGEO is primarily mediated through its capacity to disrupt bacterial cell envelopes. Studies demonstrate that LGEO penetrates and destabilizes cytoplasmic membranes, causing ion and metabolite leakage, dissipation of the proton motive force, impairment of ATP production, and cytoplasmic coagulation, ultimately leading to cell death (Ali et al. 2015; Elchaghaby et al. 2022), as depicted in Figure 3. These disruptions compromise essential cellular processes such as respiration, nutrient transport, and enzyme activity, thereby creating a hostile intracellular environment that bacteria cannot recover from. Disruption of the peptidoglycan layer has also been observed, resulting in compromised cell wall integrity and the uncontrolled release of intracellular components (Elchaghaby et al. 2022). Because Gram‐positive bacteria rely more heavily on peptidoglycan for structural rigidity, they may be particularly susceptible to this mode of action. In contrast, in Gram‐negative species, membrane destabilization is often the dominant mechanism.

The key bioactive constituents driving these effects include citral (geranial and neral), citronellal, citronellol, linalool, and geraniol, with citral and geraniol in particular showing strong membrane‐disruptive activity (Dinakarkumar et al. 2024; Du et al. 2024; Gaspar et al. 2022; Sharma et al. 2024). Citral, due to its aldehyde functional group, is highly reactive with membrane lipids and proteins, leading to irreversible structural modifications. Geraniol, an alcohol, enhances membrane fluidity and permeability, facilitating leakage of cytoplasmic contents. Minor constituents, such as linalool and citronellal, although present at lower concentrations, are believed to act synergistically with citral to intensify the antimicrobial potency. Although LGEO consistently inhibits both Gram‐positive and Gram‐negative species, variation in strain‐level sensitivity has been reported (Almuhayawi et al. 2021; Dinakarkumar et al. 2024; Weshahi et al. 2025). Such variability is often attributed to differences in cell wall architecture, efflux pump activity, or adaptive stress responses among bacterial species. Its antibacterial activity is also concentration‐dependent, with higher doses producing more pronounced inhibitory outcomes (Dinakarkumar et al. 2024; Elchaghaby et al. 2022). At sublethal concentrations, LGEO may also exert bacteriostatic effects, slowing down growth rather than inducing complete cell death, which is particularly relevant for food preservation scenarios where prolonged inhibition is desired.

Formulation advances have further enhanced its antibacterial potential. For example, incorporation of LGEO into nanoemulsions and chitosan‐based coatings has improved distribution, increased surface contact, and accelerated bacterial inhibition, particularly against *E. coli* and *S. typhimurium* (Sharma et al. 2024). Nanoemulsion systems also protect volatile components from rapid evaporation and oxidative degradation, thereby extending shelf‐life and maintaining antibacterial efficacy during storage and application. These delivery systems address challenges related to volatility and stability, while expanding potential applications in food preservation.

### 3.2 Antifungal Activity

Fungal contamination remains a persistent challenge in food preservation, contributing significantly to spoilage and the accumulation of harmful mycotoxins in various commodities. Beyond its antibacterial spectrum, LGEO has also been shown to exert strong inhibitory effects against a variety of food‐related fungi. This activity is largely associated with citral (geranial and neral), the dominant bioactive component, supported by other monoterpenes, including limonene, citronellal, β‐myrcene, linalool, and geraniol (Dong and Thuy 2019; Wani et al. 2021). Importantly, the antifungal efficacy of LGEO is not confined to a single pathway; instead, it involves multiple cellular targets that collectively impair fungal growth and viability. These mechanisms range from inhibiting mycelial extension and spore germination to profound structural and metabolic disruptions (Boukhatem et al. 2014).

**FIGURE 4:**
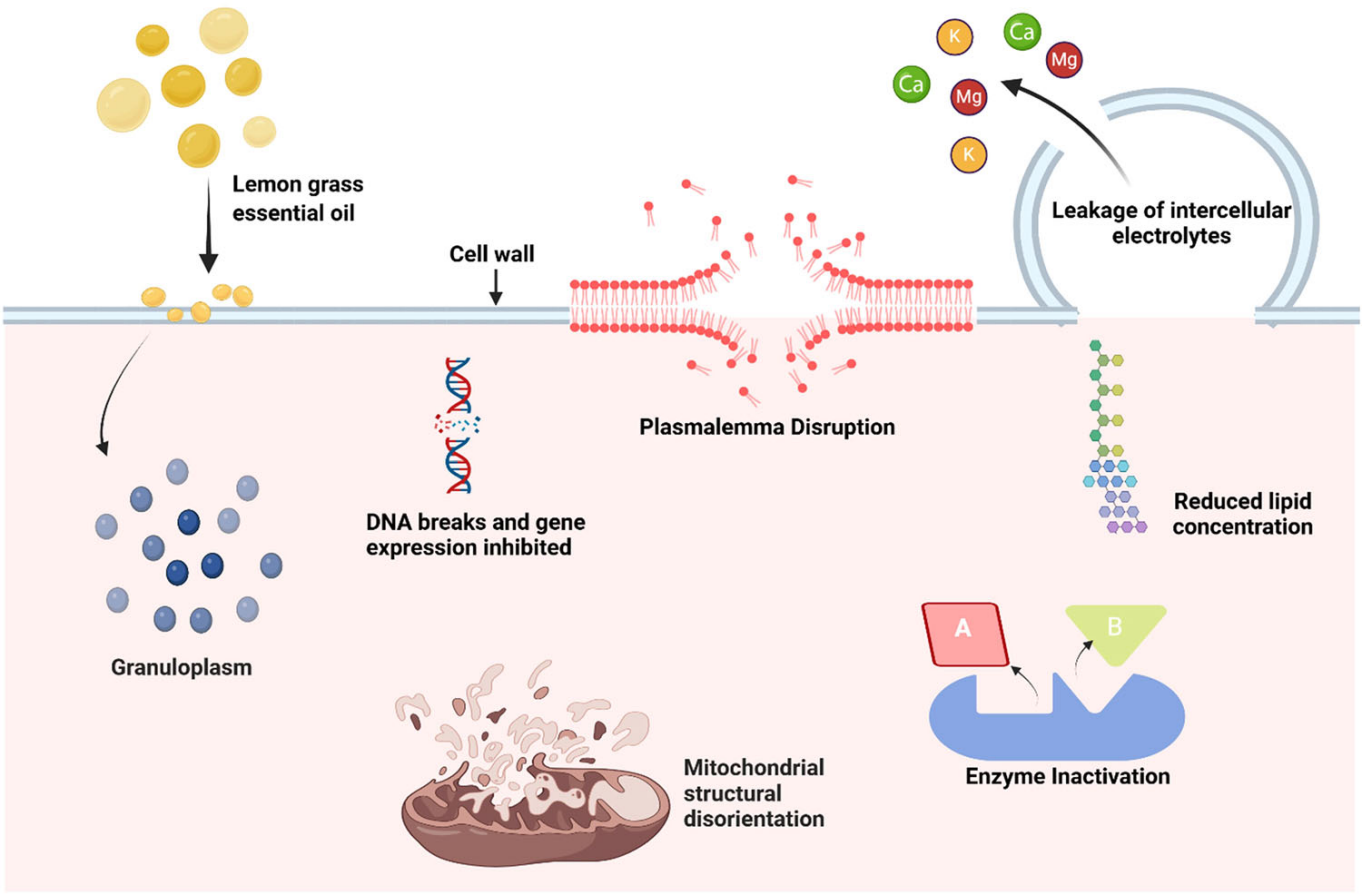
A mechanistic model for possible antifungal effect of lemongrass essential oil. Created in BioRender (https://BioRender.com/o7lwzdp).

The antifungal mechanisms of LGEO are diverse and act at multiple levels of the cell (Figure 4). One of the most widely reported effects is the inhibition of mycelial growth and spore germination, which directly reduces the ability of fungi such as *Penicillium* spp. and *Candida albicans* to propagate (Shahina and Dahms 2024). At the structural level, LGEO components penetrate and disrupt fungal cell membranes, induce mitochondrial dysfunction, and delocalize organelles, altering lipid packing and fluidity (Lee et al. 2020). This disturbance increases membrane permeability, leading to ion leakage (K⁺, Ca^2^⁺, Mg^2^⁺) and ultimately cell death. In parallel, LGEO induces oxidative stress in fungal cells, leading to the accumulation of reactive oxygen species (ROS) that compromise membrane integrity, proteins, and nucleic acids. Further damage has been linked to mitochondrial dysfunction, cytoplasmic coagulation, and impaired respiration, all of which contribute to growth inhibition and fungicidal activity (Jaouad et al. 2025).

LGEO has demonstrated inhibitory activity against a wide range of foodborne and phytopathogenic fungi. *Candida* species (e.g., *C. albicans*, *C. tropicalis*) exhibit strong susceptibility, with minimum inhibitory concentrations (MICs) often below 1 mg/mL. Conversely, filamentous fungi such as *Aspergillus flavus*, *Aspergillus niger*, and *Fusarium oxysporum* require higher concentrations, sometimes exceeding 100 mg/mL. This variability underscores the importance of tailoring concentration and delivery systems to the specific fungal target and food matrix (Majewska et al. 2019). Encouragingly, LGEO has also shown the capacity to suppress mycotoxin production by *Aspergillus* and *Penicillium*, thereby addressing not only spoilage but also direct food safety concerns associated with aflatoxins and ochratoxins (Olorunnisola et al. 2014). Experimental studies have confirmed that LGEO inhibits mycotoxin secretion by fungi, thereby preserving food grains, and it has shown strong activity against species such as *Botrytis cinerea*, *Phytophthora melonis*, *Colletotrichum coccodes*, *Phytophthora drechsleri*, *Rhizopus stolonifer*, *Cladosporium herbarum*, and *Phytophthora capsici*, making it a promising natural antifungal agent for food preservation (Amini et al. 2016; Tzortzakis and Economakis 2007). Vapor‐phase application of LGEO has been particularly effective, as volatile compounds accumulate on hyphal surfaces, intensifying antifungal action. In packaged fruits, this method has reduced *Botrytis cinerea* gray mold incidence by over 70% and offers potential for postharvest preservation (Yan et al. 2021). Zhang et al. (2022) further demonstrated that LGEO inhibited *Fusarium avenaceum* with IC_50_ values of 0.229 and 0.087 µL/mL, respectively, by disrupting plasma membrane integrity, causing protein and sugar leakage, and suppressing pectin methyl galacturonase activity, thereby reducing fungal pathogenicity. In applied food systems, LGEO demonstrated strong inhibitory effects. In yogurt, it selectively inhibited spoilage yeasts without affecting lactic acid bacteria, extending shelf life by 28 days (Milanović et al. 2021). The physicochemical properties of LGEO also enhance its antifungal efficacy. Its high volatility and lipophilicity facilitate rapid diffusion and penetration into fungal membranes, ensuring effective contact with intracellular targets (Cofelice et al. 2021). These properties make LGEO a compelling candidate for natural antifungal interventions in food systems, particularly in postharvest preservation and storage applications.

### 3.3 Antiviral Activity

In addition to its antibacterial and antifungal effects, LGEO has been reported to possess antiviral activity, although this property has been less extensively studied. The activity is thought to arise primarily from its rich content of oxygenated monoterpenes, particularly citral, citronellal, citronellol, linalool, and geraniol, which contain reactive aldehyde and alcohol functional groups capable of destabilizing lipid membranes and target multiple stages of the viral life cycle, including adsorption, transcription, and capsid stability (Adhikary et al. 2023; Devi et al. 2021). These mechanisms are particularly relevant for enveloped viruses, where disruption of the lipid bilayer can inactivate viral particles and block their entry into host cells.

Experimental evidence supports this mode of action. From a food system perspective, noroviruses represent a leading cause of gastroenteritis outbreaks and are resistant to conventional sanitizers. LGEO has been shown to effectively reduce the viral infectivity of murine norovirus (MNV), a recognized surrogate for human norovirus (Pellegrini et al. 2023). Kim et al. (2017) reported that LGEO (0.4% v/v) reduced MNV‐1 plaque formation by 75.5%, while citral (0.02% v/v) inhibited infectivity by over 70%. LGEO also suppressed human norovirus RNA replication by 58% and reduced viral shedding in mice, demonstrating preventive and therapeutic potential even under refrigeration (4°C). Supporting these findings, Bright and Gilling (2016) observed that LGEO disrupted capsid proteins of MNV‐1, reduced viral adsorption to host cells, and achieved a reduction of 1.88 log_10_ TCID_50_/mL within 6 h of exposure. Further evidence supports LGEO's activity against other norovirus surrogates such as feline calicivirus (FCV). Lanave et al. (2024) reported that LGEO caused measurable reductions in FCV within 10 min at elevated concentrations, achieving a 0.75 log_10_ decrease, an effect comparable to certain synthetic disinfectants. Similar effects are demonstrated by Pellegrini et al. (2023), who evaluated time‐ and dose‐dependent virucidal effects of LGEO against FCV, with titer reductions of up to 1.25 log_10_ TCID_50_ per 50 µL after 8 h at a concentration of 3020 µg/mL. Additional mechanistic insight was provided by Feriotto et al. (2018), who found that the LGEO compound citral reduced the infectivity of MNV by interacting with the viral capsid, forming a protective barrier that prevented host cell invasion. Overall, the findings highlight the mechanistic basis for LGEO's potential role in food system applications where norovirus contamination is a persistent hazard.

Volatile components of LGEO significantly reduced the infectivity of human mast adenoviruses, implicated in gastrointestinal and respiratory illnesses, as well as tobacco mosaic virus (TMV), with inhibition rates exceeding 50% at 100 µg/mL (Chiamenti et al. 2019). Notably, *in silico* molecular docking studies revealed that citral binds to and inhibits the main protease (Mpro) of SARS‐CoV‐2, lending mechanistic support to its broader antiviral spectrum (Thuy et al. 2021). These collective results underscore LGEO's ability to destabilize viral envelopes or interfere with adsorption to host cells, suggesting potential applications in food safety strategies, such as edible coatings, surface sanitizers, and packaging systems, to reduce viral contamination. Moreover, synergistic combinations of LGEO with natural polymers, such as chitosan, have been shown to enhance antimicrobial efficacy, and similar formulations may strengthen its antiviral potential in food preservation applications.

### 3.4 Antibiofilm Activity

Biofilm formation by foodborne and spoilage microorganisms poses an ongoing challenge in the food industry, particularly because it is associated with persistent contamination, reduced hygiene standards, and shorter product shelf life. Biofilms are highly organized microbial communities embedded within a matrix of extracellular polymeric substances (EPS), which confer mechanical stability, metabolic cooperation, and resistance to antimicrobial agents and cleaning regimens (Saharan et al. 2024). These surface‐attached communities, composed primarily of polysaccharides, proteins, lipids, and nucleic acids, form on a range of abiotic substrates, including stainless steel, plastics, and rubber, which are widely used in food processing environments. Pathogens such as *L. monocytogenes* and spoilage organisms like *Pseudomonas fluorescens* often persist in these matrices, where they can survive sanitization procedures and contribute to cross‐contamination and recurrent product recalls (Chowdhury and Anand 2023; Khan et al. 2022)

Natural compounds with quorum‐quenching and biofilm‐disrupting properties are increasingly viewed as promising alternatives to traditional disinfectants. In this context, LGEO, characterized by its high citral and other oxygenated monoterpenes, has demonstrated notable antibiofilm activity against a range of food‐relevant pathogens. The lipophilic constituents of LGEO facilitate penetration into mature biofilms, enabling disruption of microbial communication networks, suppression of EPS production, and interference with adhesion and maturation processes critical for biofilm development (Khosakueng et al. 2024; Sahal et al. 2020). Specifically, LGEO has been shown to affect bacterial quorum‐sensing pathways, leading to reduced synthesis of biofilm‐associated molecules and enhanced antimicrobial sensitivity of microbial cells. These mechanisms have been validated against multiple pathogens, including *S. aureus*, *E. coli*, *P. aeruginosa*, and *L. monocytogenes*, all of which exhibited significant reductions in biofilm formation following LGEO treatment (Kim and Kim 2021).

The application of LGEO in food system settings has been explored through various *in vitro* and *in situ* studies. Chakraborty and Dutta (2022) reported that LGEO effectively disrupted *L. monocytogenes* biofilm on stainless steel surfaces, a critical concern in cold‐chain and ready‐to‐eat food processing facilities. Similarly, Valková et al. (2022) demonstrated that LGEO (citral content: 61.5%) significantly inhibited biofilm formation by *Salmonella enteritidis* and *Pseudomonas fluorescens*, both *in vitro* and on food substrates such as bread, carrots, and celery. These findings support the potential of LGEO as a vapor‐phase preservative and active packaging agent to mitigate biofilm‐associated spoilage during storage and distribution. In another study, Khosakueng et al. (2024) found that LGEO inhibited biofilm formation by *S. aureus* isolates collected from ready‐to‐eat foods, further reinforcing its applicability in real‐world food contamination scenarios.

The efficacy of LGEO against complex biofilm systems has also been demonstrated in dual‐species models. Gao et al. (2020) showed that LGEO (0.3125% v/v) and citral (0.5% v/v) significantly disrupted *S. aureus*–*C. albicans* mixed biofilms, reducing viable cell counts from approximately 92% to 14% and 13%, respectively. CLSM imaging revealed a decrease in biofilm thickness from 22 µm to 6–7 µm, with visible disruption of the matrix architecture. These findings emphasize the oil's potential for addressing polymicrobial biofilms that commonly colonize food‐contact surfaces. Similarly, Sahal et al. (2020) reported that LGEO‐coated silicone rubber surfaces exhibited 45%–76% reductions in *C. tropicalis* biofilm biomass, with MICs and minimum fungicidal concentrations (MFCs) lower than those of other tested EOs. This study highlights the relevance of LGEO for coating hydrophobic food‐contact materials that are otherwise prone to microbial colonization.

Additional investigations have addressed LGEO's performance under industrially relevant conditions. Reis‐Teixeira et al. (2019) showed that LGEO significantly reduced *L. monocytogenes* biofilm biomass on both stainless steel and glass, although complete eradication was not achieved. Structural analyses confirmed biofilm destabilization, reinforcing LGEO's utility in settings where complete microbial elimination is difficult. Wang et al. (2017) demonstrated that strong biofilm‐forming *Salmonella enterica* isolates derived from beef trim exhibited resistance to quaternary ammonium and chlorine dioxide sanitizers, but were susceptible to LGEO when applied on stainless steel and PVC surfaces at refrigeration temperature (7°C), suggesting its suitability as a chilled‐environment sanitizer in meat processing facilities.

Fresh produce systems, often characterized by biofilm‐laden surfaces and irregular topographies, also offer opportunities for LGEO application. Sun et al. (2022) observed rapid *E. coli* 0157:H7 biofilm formation on cucumber epidermis and vascular tissues, with the EPS‐rich environment offering protective advantages to the embedded cells. Given LGEO's quorum‐sensing inhibition and matrix‐disruption mechanisms, the study underscores its potential for reducing microbial persistence on produce surfaces during postharvest handling. Moreover, multispecies biofilms, which dominate food processing environments, have been shown to exhibit greater biomass and resilience compared to monocultures. Røder et al. (2015) reported that nearly 20% of four‐species combinations isolated from meat facilities generated enhanced biofilm mass at 15°C. These findings highlight the need for antibiofilm interventions, such as LGEO, that remain effective under cold‐chain and complex community conditions. Taken together, from a food‐safety perspective, LGEO's ability to inhibit and eradicate biofilms is highly significant. Biofilm formation on food‐contact surfaces, processing equipment, and packaging materials poses a serious contamination risk. By disrupting biofilm structure and impairing microbial adhesion, LGEO presents a promising natural strategy to mitigate biofilm‐associated hazards in food systems.

### 3.5 Antioxidative Activity

**FIGURE 5:**
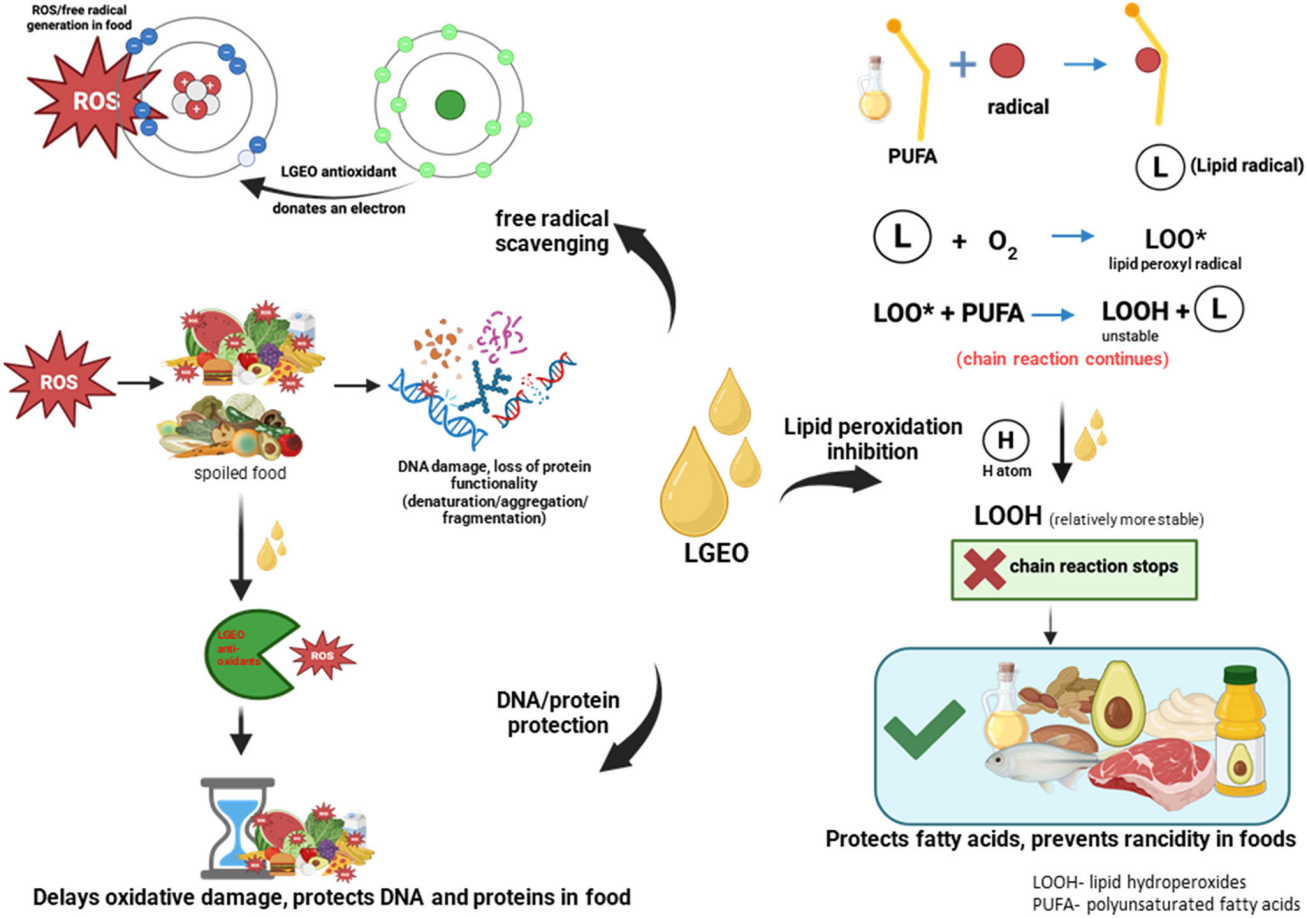
Antioxidant mechanisms of lemongrass essential oil, including free radical scavenging, inhibition of lipid peroxidation, and protection of proteins and DNA from oxidative damage. Created in BioRender (https://BioRender.com/uyxzg4g).

LGEO has emerged as a multifunctional natural additive, with antioxidant activity increasingly recognized as one of its most promising attributes for food and health‐related applications. The antioxidant potential of LGEO is closely tied to its diverse chemical profile, which primarily consists of oxygenated monoterpenes such as citral (geranial and neral), and is supported by phenolic and nonphenolic compounds, including eugenol, myrcene, linalool, limonene, and geraniol (Faheem et al. 2022). These constituents act synergistically to scavenge ROS, donate hydrogen atoms, and modulate cellular redox pathways, thereby mitigating oxidative damage to lipids, proteins, and nucleic acids (Figure 5). Such multifaceted functionality underpins the application of LGEO not only in food preservation but also in nutraceutical products aimed at counteracting oxidative stress‐associated conditions (Crespo et al. 2020; Sanches et al. 2017).

Citral is widely regarded as the principal contributor to LGEO's antioxidant capacity, with studies showing activity comparable to, or even superior to, that of synthetic antioxidants such as butylated hydroxytoluene (BHT; Kapur et al. 2016). It can regulate cytokine expression, reduce oxidative tissue damage, and provide photoprotective effects in UV‐B‐induced carcinogenesis models. Other monoterpenes such as linalool and geraniol have also demonstrated antioxidative efficacy by protecting mitochondrial function and participating in free radical scavenging, while limonene contributes to hydrogen donation and lipid oxidation inhibition (Sabogal‐Guáqueta et al. 2019).

The antioxidant strength of LGEO has been demonstrated through various assays, confirming its radical‐neutralizing capability in DPPH, ABTS, FRAP, metal chelation, and β‐carotene bleaching tests. For example, Martins et al. (2021) reported an antioxidant capacity of 22.16 ± 0.04 mg TE/g using the DPPH method, with activity increasing in a concentration‐dependent manner. Baranová et al. (2025) analyzed Nepalese LGEO and evaluated its antioxidant potential using DPPH, ABTS, and FRAP assays. LGEO exhibited moderate antioxidant activity, with DPPH IC_50_ = 5.13 mg/mL, FRAP = 3.28 mmol Fe^2^⁺ eq/g, and ABTS = 1.31 mg TE/g, indicating a notable radical‐scavenging capacity. Nevertheless, the stability of these effects is time‐limited, as citral and other volatile constituents degrade upon exposure to light and oxygen, leading to diminished antioxidant performance during prolonged storage (Faheem et al. 2022). This highlights the importance of encapsulation, controlled storage conditions, or blending with stabilizers to maintain functionality.

In food applications, LGEO's antioxidant properties are particularly valuable for delaying lipid oxidation, maintaining sensory quality, and extending shelf life (do Nascimento et al. 2023). Kieling et al. (2021) demonstrated that ethanolic extracts of LGEO effectively delayed lipid oxidation in cooked, shredded chicken breast, without affecting water activity, highlighting its potential as a natural preservative in meat systems. Reddy et al. (2023) applied LGEO (0.25%–0.5%) to ground pork and reported significantly reduced thiobarbituric acid reactive substances (TBARSs) and peroxide values over 12 days of refrigerated storage, outperforming both control and BHT‐treated groups. These findings emphasize LGEO's practical utility in extending shelf life and maintaining product quality under chilled conditions. Incorporating LGEO into different matrices has been shown to enhance the oxidative stability of fresh produce. Xiao et al. (2024) incorporated LGEO into microcapsules for coating yellow cherry tomatoes and observed 7.44% DPPH scavenging activity, which contributed to a 28‐day shelf‐life extension under refrigeration. Dietary supplementation with LGEO significantly enhanced antioxidant defense in heat‐stressed broilers as evidenced by increased superoxide dismutase (SOD) and glutathione peroxidase (GPx) activities and reduced malondialdehyde (MDA) levels, indicating strong free radical scavenging potential (Elbaz et al. 2022). Despite its potency, LGEO's antioxidant activity degrades over time, particularly due to citral instability, highlighting the importance of proper storage and formulation. Furthermore, the synergistic action of both major and minor constituents is crucial for the full expression of antioxidant activity, suggesting that whole oil preparations may be more effective than isolated compounds.

## 4 Applications in the Food System

### 4.1 Natural Preservative Functions

Foodborne pathogens and spoilage microorganisms continue to pose a significant threat to global food safety, despite advances in sanitation and processing technologies. Conventional preservatives are often synthetic, raising concerns over microbial resistance and consumer health. LGEO, due to its high bioactive content and complex phytochemistry, has emerged as a versatile natural preservative across various food systems, including fresh produce, dairy, seafood, condiments, and fermented products (Faheem et al. 2022). Its antimicrobial and antioxidant properties are increasingly harnessed through various delivery formats that address volatility, stability, and sensory constraints.

In fresh fruit preservation, inclusion complexation has proven highly effective. Phan et al. (2024) developed β‐cyclodextrin inclusion complexes of LGEO‐β‐CD and applied them to postharvest mango preservation. The treatment markedly reduced black spot incidence, improved fruit quality, and extended shelf life by 25% compared to untreated controls. In contrast, Pan et al. (2025) used LGEO in hydroxypropyl‐β‐cyclodextrin (HP‐β‐CD), achieving high antioxidant efficiency and sustained antimicrobial release. The inclusion complex significantly inhibited the growth of *E. coli* and *S. aureus*, while reducing weight loss, firmness decline, MDA accumulation, and vitamin C degradation in mangoes during 10 days of storage. This demonstrates an eco‐friendly, slow‐release preservation system that extends shelf life and maintains fruit quality in postharvest supply chains. Similarly, Debonne et al. (2022) compared the antifungal activity of clove, lemongrass, and thyme EOs for natural preservation of dried apricots, finding that LGEO inhibited *Eurotium* spp. at ∼0.25% in microdilution assays, supporting its role as a natural alternative to sulfite fumigation in dried fruit preservation. Thakur et al. (2023) reported that treatment with 5 µL/L LGEO extended the vase life of *Gladiolus grandiflorus* cut spikes from 9 to 11 days by reducing microbial blockage, enhancing antioxidant enzyme activity, maintaining sugar and carotenoid levels, and downregulating senescence‐associated genes.

Seafood systems have been a strong focus for LGEO delivery and preservative innovations. Zhou et al. (2023) demonstrated that combining LGEO (0.008 g/mL) with tea polyphenols enhanced the antioxidant capacity and antibacterial effects *in vitro*. When applied to marine fish fillets. This combination reduced microbial counts, suppressed biogenic amine accumulation, and delayed flavor deterioration. Yunilawati et al. (2023) developed antibacterial sachets containing β‐cyclodextrin/LGEO inclusion complexes and applied them to shrimp packaging at room temperature. The sachets achieved high encapsulation efficiency, provided sustained release of citral and geraniol, inhibited *E. coli* and *S. aureus*, and delayed spoilage by lowering total volatile basic nitrogen (TVBN) levels. The shelf life was extended by 12 h compared to the controls. Qian et al. (2025) used LGEO oil in β‐cyclodextrin inclusion complexes and incorporated the complexes into sustained‐release absorbent pads to preserve large yellow croaker fillets. Compared with free LGEO, the LGEO‐IC pads exhibited stronger antibacterial activity against *E. coli*, *S. aureus*, and *Shewanella putrefaciens*, reduced total viable counts, and maintained pH, texture, and water‐holding capacity during 10 days of cold storage, highlighting their potential as scalable natural preservative systems for seafood.

In dairy systems, LGEO has shown strong natural preservative potential. Abed et al. (2022) evaluated LGEO as a preservative in yogurt. They found that a concentration of 125–500 ppm significantly inhibited coliforms, yeasts, and molds, with 500 ppm achieving up to 100% inhibition of fungal growth. Yogurt treated with LGEO showed delayed spoilage for up to 6 weeks at 25°C and 3 months at 5°C. Al‐Hamdani et al. (2024) found that adding 100–200 mg/L lemongrass extract during ultrafiltration concentration of milk improved microbial safety and significantly reduced total bacterial counts while enhancing protein and total solids, suggesting compatibility with modern processing technologies. Al‐Hamdani et al. (2024) applied LGEO extract as a preservative during milk concentration using ultrafiltration and observed that additions of 100–200 mg/L significantly reduced total bacterial counts while improving protein and total solids content in the concentrate. The study demonstrated that lemongrass can enhance microbial safety and quality of concentrated milk.

Condiments, sauces, and silage also benefit from LGEO's natural preservative efficacy. Hanková et al. (2023) demonstrated that LGEO at 512 mg/kg reduced *E. coli* counts by 1.9 log CFU/g within 5 days and below detectable levels by Day 15, comparable to citric acid and heat treatment. Importantly, sensory evaluation demonstrated that LGEO‐treated salsa retained its acceptable aroma and taste, thereby validating its role in ensuring the safety of ready‐to‐eat products. Fermented products represent another application, as Saliu et al. (2024) synthesized silver nanoparticles using lemongrass extract (LSSNP) and applied them to fermented locust beans (*Parkia biglobosa*), achieving a 63.7% reduction in microbial load and preserving sensory and proximate qualities during storage. The treatment inhibited spoilage bacteria, including *Bacillus* and *Enterobacter* spp., while maintaining safety in animal studies, demonstrating LSSNP as a green, sustainable preservation strategy for extending the shelf life of traditional fermented condiments. Del Valle et al. (2025) investigated the use of LGEO (2 mL/kg) in rehydrated corn silage combined with heterofermentative inoculants (*Lentilactobacillus buchneri* and *L. hilgardii*). While microbial inoculation altered fermentation dynamics, the addition of LGEO alone did not significantly influence fermentation profile, nutrient recovery, or aerobic stability, indicating that under high‐dry‐matter silage conditions, LGEO provides limited preservative benefit compared with its stronger effects reported in other food systems.

### 4.2 Incorporation Into Edible Films and Coatings

Edible films incorporating LGEO have gained prominence as sustainable packaging solutions because they combine physical barrier properties with bioactive preservation. Such systems reduce moisture loss, limit oxygen diffusion, and inhibit microbial growth, thereby extending the shelf life of perishable foods (Table 2). In addition to protecting quality, LGEO‐based films can enhance sensory and nutritional attributes by preserving aroma, color, and antioxidant activity (Manjunatha et al. 2023). Several studies confirm the efficacy of LGEO in animal‐derived foods. Aitboulahsen et al. (2020) developed gelatin–pectin films incorporating, an EO, for tilapia fillets stored at 4°C. The films enriched with 1.5% LGEO significantly reduced total viable counts, lactic acid bacteria, and *Enterobacteriaceae*, while lowering TVB‐N values and doubling shelf life from 6 to 12 days. Similarly, Azizah et al. (2023) demonstrated that fish gelatin–pectin composite films with LGEO enhanced mechanical properties, improved antioxidant and antibacterial activity, and maintained the quality of chicken breast under refrigeration. Bhatia et al. (2024) further developed pectin–sodium caseinate films impregnated with LGEO, which increased antioxidant activity from 16.6% to 40.4%, imparted antibacterial activity against *E. coli*, and improved gloss and hydrophobicity, thereby reinforcing their suitability as active edible packaging for meat applications. Notably, alginate films coloaded with silver nanoparticles and LGEO extended the shelf life of cheese for 14 days by suppressing the growth of *E. coli*, *S. aureus*, *Salmonella*, and *B. cereus*, suggesting that combining LGEO with nanomaterials can enhance antimicrobial activity in dairy systems (Motelica et al. 2021).

**TABLE 2:** Application of lemongrass essential oil‐based coatings and films in food preservation and shelf‐life extension.

Coating material	Food product targeted	Microorganism targeted	Procedure	Shelf‐life extension	References
LGEO (β‐CD/LO) Inclusion Complex	Shrimp	*Escherichia coli* and *Staphylococcus aureus*	The β‐CD/LO inclusion complex was prepared by coprecipitation, loaded into nonwoven fabric sachets, and placed in shrimp packaging at room temperature (28 ± 2°C).	Extended shrimp freshness by 12 h at room temperature by reducing bacterial spoilage, monitored through total volatile base nitrogen (TVBN) values.	Yunilawati et al. (2023)
LGEO + alginate	Fresh‐cut pineapple	Yeasts, molds, total plate count (TPC)	Pineapple cubes were coated with alginate‐based edible coatings containing different concentrations of LGEO and stored at 10 ± 1°C for 16 days.	LGEO at 0.3% (w/v) was most effective, extending microbial shelf‐life to 12 days, while 0.5% (w/v) extended it to 16 days. However, higher LGEO concentrations reduced texture and sensory quality.	Azarakhsh et al. (2014)
LGEO	Fresh orange juice	*Escherichia coli*, *Salmonella typhi*, *Bacillus subtilis*, *Staphylococcus aureus*, *Saccharomyces cerevisiae*, *Aspergillus niger*, *Aspergillus flavus*	Fresh orange juice was treated with varying concentrations of lemongrass essential oil (0.5, 0.75, 1, and 1.25 µL/mL). Samples were stored at 4°C and analyzed for microbial load, physicochemical stability, and antioxidant properties.	The use of 1.25 µL/mL LGEO resulted in the highest microbial inhibition, significantly reducing the growth of bacteria, fungi, and yeast, and improving shelf life up to 12 days. Lower concentrations showed moderate effects.	Abou‐Raya et al. (2023)
LGEO + modified atmosphere packaging (MAP)	Strawberries	Fungal spoilage organisms, bacteria causing fruit rot	Strawberries were immersed in 0.2% Lemongrass Oil and stored at 4.0 ± 1.0°C under modified atmosphere packaging (MAP). The fruit storage duration was monitored for 21 days.	MAP + LGEO extended shelf life to 18 days (“Sahara”), 15 days (“Florida Fortuna,” “Rubygem”), and 12 days (“Camarosa”). Control fruits lasted only 2–5 days.	Kahramanoğlu (2019)
LGEO + carnauba‐shellac wax (CSW) nanoemulsion	“Fuji” apples	*Escherichia coli*, *Listeria monocytogenes*, yeast and molds	Fresh apples were dipped in CSW–LGEO solution for 30 s and dried before storage at 1°C for 5 months. Apples were analyzed for microbial load, weight loss, texture, and sensory properties.	Apples coated with CSW–LGEO maintained hardness, reduced weight loss (5.2% vs. 7.7%), and had no yeast or mold growth detected after 5 months of storage. Sensory properties were also well preserved.	Jo et al. (2014)
LGEO incorporated into gelatin–pectin‐based film	Tilapia fillets	Psychrotrophic bacteria, total viable count (TVC), lactic acid bacteria (LAB), enterobacteriaceae (ENT)	Edible biodegradable films were prepared by incorporating LGEO into gelatin/pectin‐based coating. Fish fillets were coated and refrigerated.	Shelf life extended to 12 days by reducing microbial load and maintaining physicochemical quality.	Aitboulahsen et al. (2020)
LGEO nanoemulsion + chitosan	Grape berries	*Salmonella typhimurium*, Total mesophilic aerobes, yeasts, and molds	Nanoemulsion formed using dynamic high‐pressure processing (DHP) and high‐shear mixing (HSM), followed by dip‐coating of grape berries.	An extended shelf‐life of grape berries up to 28 days at 4°C and up to 14 days at 25°C, with reduced microbial growth and quality deterioration.	Oh et al. (2017)
LGEO + arabic gum (AG) and maltodextrin (MD) microcapsules	Coalho cheese	*Escherichia coli*, *Staphylococcus aureus*, Total coliforms	Microencapsulation via spray drying, using an inlet temperature of 170°C and a feed flow rate of 0.9 L/h.	Microencapsulated LGEO (AG/MD 3:1) was the most effective, extending shelf life beyond 21 days. AG/MD (1:1) was slightly less effective. Nonmicroencapsulated LGEO showed antimicrobial effects but lost efficiency over time due to volatilization.	Melo et al. (2020)
LGEO	Rice	*Aspergillus flavus (aflatoxigenic fungi)*	Direct application as fumigant and incorporation in liquid media.	Lemongrass oil was fungistatic at 0.6 mg/mL and fungicidal at 1.0 mg/mL. It inhibited aflatoxin production at 0.1 mg/mL, suggesting a significant improvement in storage safety.	Paranagama et al. (2003)
Microemulsion containing LGEO and oregano with citrus extracts	Orange juice	*Aspergillus niger, Penicillium chrysogenum, Saccharomyces cerevisiae*	Microfluidization to prepare an oil‐in‐water microemulsion, followed by γ‐irradiation treatment (1 kGy).	The combination of microemulsion and γ‐irradiation completely eradicated fungal and yeast growth, maintaining microbiological stability for 35 days at 4°C.	Maherani et al. (2019)
LGEO	Buffalo milk yogurt	*Debaryomyces hansenii, Candida pararugosa, Yarrowia deformans*	Direct incorporation in the yogurt formulation and evaluation in an *in vivo* yogurt model.	Lemongrass EO completely inhibited D. hansenii and Y. deformans while reducing the growth of C. pararugosa. The yogurt remained microbiologically stable for 28 days at 4°C.	Milanović et al. (2021)
Starch/alginate‐based bioactive films + LGEO	Active food packaging	*Staphylococcus aureus*, *Escherichia coli*	Film formation via the solution casting method, incorporating different concentrations of lemongrass essential oil.	Lemongrass essential oil films exhibited bacteriostatic activity against *S. aureus* and *E. coli* and demonstrated extended antimicrobial stability, suggesting potential for prolonged shelf life in packaged foods.	Vargas et al. (2024)
LGEO + thermal treatment (80°C, 30–90 s)	Mixed fruit juices	*Saccharomyces cerevisiae*	Direct incorporation at MIC (1.13 mg/mL), ½ MIC (0.56 mg/mL), and ¼ MIC (0.28 mg/mL), combined with thermal treatment.	Complete growth inhibition at the MIC level; combination with thermal treatment reduced the required oil concentration and extended juice stability up to 7 days.	Tyagi et al. (2014)
LGEO + chitosan‐starch film	Various food packaging applications	*Escherichia coli*, *Salmonella typhi*, *Staphylococcus aureus*, *Staphylococcus epidermidis*	LGEO was incorporated into chitosan‐starch film as an active packaging material to enhance antimicrobial and antioxidant activity.	Improved antimicrobial properties and enhanced food safety, indicating extended shelf life.	Istiqomah et al. (2022)
LGEO (1 µL/g)	Cream‐filled cakes and pastries	*Escherichia coli*, *Salmonella typhimurium*, *Candida albicans*, *Bacillus cereus*	Direct incorporation into the cake cream filling, followed by baking at 120°C for 10 min.	More than 99.9% reduction in microbial counts and no observable microbial growth after 72 h of storage.	Vazirian et al. (2012)
Arabic gum + sodium caseinate + LGEO	Guava	Various spoilage fungi and bacteria	Dipping method: Guavas were dipped in edible coating solution for 5 min, air‐dried, and stored at 4–7°C for 35 days, followed by ripening at 25°C for 5 days.	Extended up to 40 days compared to 7 days for uncoated guavas.	Murmu and Mishra (2018)
Mesoporous silica (MSNPs) nanoparticles loaded with LGEO+ clove oil + sodium alginate coating	Wheat seeds	*Gaeumannomyces graminis var. tritici*	Encapsulation of essential oils into MSNPs via the sol‐gel method, followed by seed treatment with alginate‐based nanoformulation.	Disease control improved to 84% with alginate‐coated MSNPs, prolonging antifungal activity compared to free EOs, which achieved only a 57.44% control rate.	Sattary et al. (2020)
Gum arabic (GA) + maize starch (MS) + LGEO	Pomegranate fruit	Various spoilage bacteria and fungi	Dipping method: Fruits submerged in coating solution for 1 min, air‐dried, stored at 5 ± 1°C for 42 days, and evaluated after 5 days at ambient temperature (20 ± 0.2°C).	Extended up to 47 days compared to uncoated fruits.	Kawhena et al. (2021)
Nanoemulsion of clove oil (CO) and LGEO	Tomato	*Fusarium oxysporum* f.sp. *lycopersici* (tomato wilt pathogen)	Nanoemulsion prepared via low‐energy emulsification method, tested as a soil amendment and seed/seedling treatment.	Wilt disease incidence reduced by 70.6% in tomato plants, indicating enhanced protection and extended plant survival.	Sharma et al. (2018)
LGEO + chitosan film	Active food packaging	*Bacillus cereus, Escherichia coli*, *Listeria monocytogenes*, *Salmonella typh*	Chitosan‐based films were incorporated with LGEO at varying concentrations (1%–9%) and tested for antimicrobial properties using the Kirby–Bauer disk–diffusion method.	Chitosan films with 9% LGEO exhibited the highest antimicrobial activity, preventing bacterial growth and improving food safety. Suitable for enhancing shelf‐life in food packaging.	Han Lyn and Nur Hanani (2020)
Microemulsion of LGEO	Iceberg lettuce	*Salmonella enterica* and *Lactobacillus casei*	Washing treatment with antimicrobial microemulsion, followed by refrigeration at 4°C for 28 days.	Up to 28 days with significant bacterial reduction (2.3–4.37 log CFU/g for Salmonella and 0.11–4.25 log CFU/g for Lactobacillus).	Arellano et al. (2021)
Chitosan + LGEO	Guava, mango, and papaya	*Colletotrichum asianum, Colletotrichum fructicola, Colletotrichum tropicale, Colletotrichum siamense, Colletotrichum karstii*	Fruits were coated by immersion in chitosan–LGEO dispersions, air‐dried, and stored at 25°C for 12 days.	Up to 12 days, with significant inhibition of anthracnose lesion development (100% reduction in some cases) compared to synthetic fungicides.	Lima Oliveira et al. (2018)
Gelatin film incorporated with LGEO	Sea bass slices	Lactic acid bacteria, psychrophilic bacteria, and spoilage microorganisms (*Enterobacteriaceae*)	Slices were wrapped with gelatin films containing LGEO and stored at 4°C for 12 days.	Extended up to 12 days compared to uncoated control samples, significantly inhibiting microbial growth and delaying spoilage.	Ahmad et al. (2012)

Plant‐based films incorporating LGEO have also shown promising preservation effects. Kaushani et al. (2025) demonstrated that alginate‐based edible films enriched with up to 1.5% LGEO improved water resistance, antioxidant activity, and antimicrobial efficacy against *S. aureus*, *B. cereus*, *K. pneumoniae*, and *E. coli*, while maintaining the quality and microbial safety of minimally processed carrots under refrigeration. Chitosan–velvet bean extract films with 3% LGEO significantly enhanced antibacterial activity against *S. aureus* and *E. coli* and improved antioxidant performance in fruit systems (Ariani et al. 2025). In starch‐based matrices, LGEO improved antioxidant activity but often compromised mechanical strength: sweet potato starch films containing LGEO increased elongation at break to 71.8% and provided 29%–43% DPPH scavenging activity, but tensile strength was reduced (Kadir Basha et al. 2020). Sago starch films (1%–5% LGEO) also inhibited microbial growth. However, higher oil loads increased water vapor permeability, again demonstrating the trade‐off between antimicrobial potency and barrier/mechanical performance (Santosa et al. 2019). Seaweed hydrocolloid–GAC pulp (*Momordica cochinchinensis*) films with LGEO exhibited improved moisture barrier properties and elongation without compromising transparency, indicating potential application for LGEO in bakery or plant‐based food preservation (Tran et al. 2021). Apple pectin–cellulose nanocrystal films with LGEO reduced weight loss and delayed anthocyanin accumulation in strawberries, while chitosan–pectin films incorporating LGEO prolonged raspberry shelf life from 4 to 8 days (Jovanović et al. 2021). Collectively, these results suggest that LGEO is highly effective at extending the shelf life of fruits, vegetables, and bakery items. Beyond single‐function preservation, LGEO has been explored in multifunctional packaging. Polyvinyl alcohol–starch films embedding LGEO via β‐cyclodextrin inclusion complexes exhibited potent antioxidant activity. Additionally, it inhibited the growth of *Shewanella putrefaciens* while maintaining barrier integrity, demonstrating promise for use in aquatic food packaging (Chen et al. 2020).

While edible films provide standalone protective layers that can be applied to foods or used as wraps, their functional principles closely overlap with those of edible coatings. The main distinction lies in the mode of application: films are preformed sheets, whereas coatings are directly applied as thin layers that conform to the surface of the food. This difference makes coatings particularly attractive for fresh produce and bakery items, where a seamless barrier is required without altering product appearance or texture. Building on the demonstrated advantages of LGEO‐incorporated films, the use of LGEO in edible coatings has attracted increasing attention as an eco‐friendly postharvest preservation strategy.

A substantial body of work confirms the broad utility of LGEO coatings in extending the storage life of fresh produce. Thi Hang Phuong et al. (2024) demonstrated that chitosan–LGEO coatings reduced weight loss, maintained firmness and pH, and preserved color in strawberries for 24 days at 2°C. Importantly, X‐ray CT scans revealed enhanced tissue integrity, confirming structural protection beyond superficial quality. Bansal et al. (2024) showed similar benefits in plums using buckwheat starch–xanthan gum coatings enriched with 1.25% LGEO, which provided inhibition zones of 22.1 mm (*E. coli*) and 28.7 mm (*S. aureus*) and displayed 73.3% DPPH radical scavenging activity. These results link antimicrobial action with antioxidant capacity, delaying weight loss and shrinkage over 20 days of cold storage. Similarly, Mishra et al. (2018) highlighted the long‐term bioactivity potential of cellulose nanofiber–polyethylene glycol–LGEO composites, where citral release followed pseudo‐Fickian diffusion for up to 120 days, demonstrating the feasibility of coatings designed for prolonged activity during transport and retail distribution. In another study, Irianto et al. (2021) demonstrated that alginate‐based coatings with 2.5%–7.5% LGEO exhibited antibacterial activity against *E. coli*, *S. aureus*, *Salmonella*, and *P. aeruginosa*, with maximum inhibition at 5% LGEO, suggesting broad‐spectrum applications across animal foods.

In other fruit applications, Erceg et al. (2023) incorporated LGEO into a chitosan–gelatin matrix using β‐cyclodextrin complexes, completely suppressing *Penicillium aurantiogriseum* and extending cherry tomato shelf life to 20 days. Similarly, de Oliveira et al. (2020) reported that a chitosan–LGEO composite (0.6 µL/mL) delayed guava ripening by stabilizing firmness and pH while improving consumer acceptance. Almeida et al. (2024) demonstrated that cassava starch–LGEO coatings reduced weight loss by 83.7%, maintained chlorophyll, and completely prevented anthracnose symptoms in “Palmer” mangoes, a result highly relevant to commercial mango exporters. Yadav et al. (2025a) extended this application to guavas, where 1% LGEO coatings increased guava shelf life from 9 to 21 days under cold storage, while retaining firmness, antioxidant activity, and sensory acceptance . In strawberries, da Silva et al. (2019) showed that apple pectin–cellulose nanocrystal coatings enriched with LGEO not only reduced weight loss but also delayed anthocyanin accumulation, preserving both nutritional and visual quality. At ambient conditions, Yadav et al. (2025b) reported that 1% LGEO in mango kernel starch coatings reduced weight loss, respiration, and ethylene production, maintained bioactive compounds and firmness, and extended tomato shelf life to 15 days at room temperature and 30 days under cold storage.

Likewise, Toazza et al. (2021) found that coatings containing *C. citratus* and *C. flexuosus* EOs preserved phenolic content, reduced fungal decay, and maintained strawberry quality for 15 days. Bajaj et al. (2024) reported that guar gum–LGEO composites applied to Kinnow mandarins reduced spoilage (3.01%), weight loss (7.21%), and lipid peroxidation, while maintaining higher ascorbic acid and flavonoid levels and extending storage life to 75 days under refrigeration, performance highly competitive with synthetic preservatives. Kawhena et al. (2022) reinforced this by demonstrating gum arabic–LGEO coatings, with or without pomegranate peel extract, reduced decay and respiration in pomegranates during 6 weeks of cold storage. Together, these studies highlight LGEO's versatility across diverse fruit systems, including berries, tomatoes, tropical fruits, and citrus.

The application of LGEO coatings has also been extended to bakery systems, where mold growth is a significant constraint on shelf life. Bautista‐Espinoza et al. (2023) formulated quinoa protein–chitosan coatings reinforced with silica nanoparticles encapsulating LGEO and cinnamon oil. These coatings exhibited strong antifungal activity against *Rhizopus stolonifer*, a typical bread contaminant, and extended the shelf life of sourdough bread by 3.5 days without compromising sensory quality. This study is particularly relevant for industrial bakery chains, where a short shelf life contributes significantly to food waste.

Cutting‐edge formulations integrate LGEO with nanostructures or additional actives to achieve multifunctionality. In a study, Gago et al. (2022) demonstrated that alginate–LGEO coatings (1.25%) delayed superficial scald and maintained pear sensory quality for up to 6 months, achieving efficacy comparable to that of 1‐MCP, the commercial gold standard. This finding strongly underscores the industrial viability of LGEO coatings in high‐value fruit storage chains. Despite these promising outcomes, limitations remain. Hirsch et al. (2024) reported that LGEO was less effective than oregano or thyme oils in gelatin coatings, with limited long‐term protection against *Bacillus subtilis*. This highlights a recurring challenge: while LGEO offers broad antimicrobial and antioxidant activity, its efficacy may be weaker than that of some other EOs, particularly when stored for an extended period. Moreover, high LGEO concentrations can compromise mechanical performance, increase water vapor permeability, and cause intense aromas that affect consumer acceptability (Ghamari et al. 2022). Another critical gap is the lack of pilot‐scale and real supply‐chain validation, as most studies remain confined to laboratory or short storage trials.

### 4.3 Active and Intelligent Packaging

LGEO has emerged as a promising bioactive ingredient for the design of active and intelligent food packaging systems, thanks to its multifunctional properties (Table 3). Active packaging refers to systems that interact directly with the food or its environment to extend shelf life. In contrast, intelligent packaging is designed to monitor and signal changes in food quality (Mkhari et al. 2025). To fabricate active packaging, LGEO is commonly incorporated into biodegradable matrices such as chitosan, starch, cellulose, alginate, or paperboard, where it can be released in a controlled manner to inhibit microbial growth and oxidative processes (Casalini and Giacinti Baschetti 2023). For instance, Silva et al. (2025) successfully coated paperboard with a chitosan–LGEO emulsion, producing packaging films with measurable antimicrobial activity, enhanced mechanical stiffness and hydrophobic barrier properties, and insecticidal activity against grain pests. Cytotoxicity assays confirmed their safety for food contact.

**TABLE 3:** Recent studies on LGEO applications in active and intelligent packaging.

Biopolymer used	Conc. of LGEO	Bioactivity	Delivery method	References
Gelatin/pectin	0.5% and 1.5% (v/v)	Antimicrobial against *Listeria monocytogenes*, *E. coli*, *S. aureus*, *S. typhimurium*, *Enterococcus faecalis*, and *Pseudomonas aeruginosa*	Solution casting	Aitboulahsen et al. (2020)
Fish gelatin–pectin composite	0.5% (v/v)	Antimicrobial against *Salmonella* and antioxidant, DPPH radical scavenging up to 24.2%	Solution casting	Azizah et al. (2023)
Chitosan	1%–5% (v/v)	Antimicrobial activity against *Escherichia coli* and *Staphylococcus aureus* and antioxidant activity (DPPH scavenging ≈ 83%)	3D printing	Li et al. (2022b)
Pectin–sodium caseinate	0.1%, 0.2%, and 0.3% (v/v)	Antimicrobial against *E. coli* and antioxidant up to 40.41% (DPPH assay)	Solution casting	Bhatia et al. (2024)
Chitosan–paperboard composite	20%, 30%, and 40% (w/v)	Antimicrobial and antioxidant	Emulsion coating	Silva et al. (2025)
Chitosan	0.5%, 1.5%, and 2.5% (v/v)	Antimicrobial effects against *E. coli* and *S. aureus* and antioxidant (ORAC = 495.4 µmol TE/g)	Solution casting	Contini et al. (2022)
Sodium alginate	1%–5% (w/w)	Antifungal activity against *Aspergillus niger* and *Botrytis cinerea*	3D printing	Li et al. (2022b)
Cellulose acetate (CA)/poly(caprolactone diol) (PCL‐diol)	3%, 5%, and 7 wt%	Antimicrobial activity against *S. aureus*, *L. monocytogenes*, *E. coli*, and *Salmonella Typhimurium*	Solution casting	Erceg et al. (2022)
Chitosan	1, 3, 5, 7, and 9 wt%	Antimicrobial activity against *Bacillus cereus*, *E. coli*, *Listeria monocytogenes*, *Salmonella typhi*; and *S. typhi*	Solution casting	Han Lyn and Nur Hanani (2020)
Corn starch with montmorillonite (MMT) and starch nanocrystals (SNC)	2% (v/v)	Antimicrobial activity against *E. coli* and *S. aureus* and antioxidant activity (DPPH = 76.2%)	Solution casting	Kaur et al. (2025)
Gelatin/palm wax	12% (w/w)	Antimicrobial efficacy against *Staphylococcus aureus*, *Listeria monocytogenes*, *Escherichia coli*, and *Salmonella Typhimurium* and antioxidant activity (DPPH = 79.21 ± 0.17%)	Coating	Nurul Syahida et al. (2021)

Similar approaches using polysaccharide matrices have demonstrated that LGEO addition reduces water vapor permeability and enhances mechanical strength. Contini et al. (2022) developed antioxidant chitosan films containing 2.5% LGEO, which reduced water vapor permeability and total soluble matter, improved lipid oxidation stability in chicken patties, and maintained microbiological quality under refrigeration, though sensory acceptance declined due to LGEO's strong odor and flavor. LGEO's intelligent packaging applications are gaining traction, as its volatile components can serve as indicators of spoilage and quality changes. Erceg et al. (2022) developed biodegradable cellulose acetate–poly(caprolactone diol) active packaging plasticized with glycerol tritartarate and enriched with up to 7% LGEO, which improved tensile strength, torsion resistance, and antimicrobial efficacy against *S. aureus*, *L. monocytogenes*, yeasts, and fungi, while effectively reducing fungal spoilage on grapefruit slices and extending storage life by more than twofold. Encapsulation strategies further enhance LGEO's suitability for intelligent packaging. Han Lyn and Nur Hanan (2020) developed chitosan films for active packaging incorporating up to 9% LGEO, which improved elongation at break by 101%, reduced water vapor permeability by 15%, and exhibited strong antimicrobial activity against *B. cereus*, *E. coli*, *L. monocytogenes*, and *S. typhi*. Kaur et al. (2025) showed that starch‐based nanocomposite active films reinforced with starch nanocrystals or montmorillonite and loaded with 2% LGEO nanoemulsion improved tensile strength, reduced water vapor transmission, and provided strong antimicrobial and antioxidant activity, effectively extending the shelf life of cottage cheese from 4 days (unpackaged) to 12 days under refrigeration. More works are extending active packaging applications to meat products. Nurul Syahida et al. (2021) showed that Kraft paper coated with gelatin, palm wax, and 12% LGEO significantly reduced microbial growth, lipid oxidation, pH, and color changes in ground beef stored at 4°C, maintaining bacterial counts below the permissible limit for 7 days compared to 5 days in controls, thereby extending shelf life and confirming its potential as an active paper‐based packaging material for meat products. Li et al. (2022b) fabricated 3D‐printed sandwich‐like chitosan–anthocyanin–LGEO films that combined antioxidant and antibacterial activity with pH‐sensitive colorimetric response, enabling smartphone‐based monitoring of pork freshness while extending shelf life under refrigeration, thus demonstrating their potential as intelligent active packaging systems for meat products. Such systems align with current industrial trends in smart packaging, where consumer demand is driving the development of films and coatings that both extend shelf life and provide transparency regarding food quality. Despite promising results, LGEO‐based active packaging faces key limitations, including variability in oil composition, strong aroma affecting consumer acceptance, and limited data on stability and migration in real food systems.

### 4.4 Emulsion‐Based Formulation

#### 4.4.1 Nanoemulsions

Nanoemulsions represent a significant advancement in food preservation, offering enhanced solubility, stability, and bioactivity of EOs. By reducing particle size to the nanoscale (20–200 nanometers), nanoemulsions enhance the dispersion of active compounds in the food matrix, thereby protecting them from degradation (Niroula et al. 2024). For LGEO, these attributes are particularly valuable, as citral and other monoterpenes are prone to oxidation, volatility, and strong aroma release. Encapsulation in nanoemulsions not only enhances antimicrobial and antioxidant efficacy but also mitigates the release of off‐flavors, thereby offering greater consumer acceptability in real food applications (Ben‐Fadhel et al. 2024).

Several studies have demonstrated the potent antimicrobial activity of LGEO nanoemulsions in perishable foods, Singh et al. (2023) showed that corn starch films reinforced with montmorillonite (1.5%) or starch nanocrystals (1%) and loaded with 2% LGEO nanoemulsions significantly reduced weight loss, microbial growth, and decay in strawberries, while preserving firmness, phenolics, anthocyanins, and antioxidant enzyme activity, thereby extending shelf life from 4 days (control) to 8 days under refrigeration. Similarly, Oh et al. (2017) demonstrated that grape berries coated with chitosan–LGEO emulsions produced by dynamic high‐pressure processing (∼232 nm) had greater inhibition of *S. typhimurium*, mesophilic aerobes, yeasts, and molds, while retaining color, soluble solids, and antioxidant activity more effectively than high‐shear‐prepared emulsions. Putra et al. (2025) further confirmed the value of LGEO nanoemulsions in bananas, reporting that chitosan‐encapsulated nanoemulsions (70–135 nm) exhibited strong antioxidant activity (IC50 31 ppm), suppressed *B. cereus* and *E. coli*, and delayed ripening while maintaining firmness and color for up to 11 days. These studies collectively highlight the potential of LGEO nanoemulsions as edible coatings that can extend the storage life of high‐value fruits.

The performance of nanoemulsions is significantly influenced by the preparation techniques and stabilizers, which determine the droplet size, stability, and release behavior. Hebishy et al. (2022) demonstrated that high‐intensity ultrasound combined with food‐grade emulsifiers, such as soy lecithin, whey protein hydrolysates, or gum arabic, yielded nanoemulsions with enhanced physical, thermal, and oxidative stability while maintaining antimicrobial activity against *S. aureus*, *L. monocytogenes*, and *E. coli*. Rabbani et al. (2025) compared premix membrane emulsification, ultrasonication, and high‐pressure homogenization, demonstrating that LGEO‐in‐water nanoemulsions (0.1–10 µm) retained their antimicrobial and antioxidant properties across all methods. Shelf‐life modeling predicted activity of these nanoemulsions of up to 115 days at 4°C and 60 days at room temperature. Similarly, Mohd Daud et al. (2025) reported that supercritical fluid‐extracted LGEO nanoemulsions (∼86 nm) achieved near‐complete suppression of *B. cereus* isolates at 2% concentration for up to 4 months and maintained thermal stability over 21–90°C. However, droplet growth and a reduction in zeta potential during storage highlighted the need for improved stabilization strategies. Complementing these findings, Liu et al. (2023) showed that LGEO nanoemulsions stabilized with Tween 80 and TEMPO‐oxidized cellulose nanofibrils (∼19 nm) achieved strong thermodynamic and freeze–thaw stability for 30 days and enhanced antifungal activity against *A. flavus* compared to pure LGEO.

Beyond their antimicrobial role, LGEO nanoemulsions have also been tailored for multifunctional and nanocomposite systems. Cofelice et al. (2021) demonstrated that alginate‐based nanoemulsions containing 1.5% LGEO inhibited *Rhizopus* spp., *Penicillium expansum*, and *Aspergillus niger* for up to 10 days, while maintaining rheological stability, indicating their suitability as antifungal coatings. Liu et al. (2024) extended their use to grain storage, showing that LGEO nanoemulsions (∼410 nm) loaded with SiO_2_ nanoparticles (57.45% capacity) enhanced acaricidal activity against *Aleuroglyphus ovatus*, achieving higher mortality, ovicidal inhibition, and repellency compared to pure LGEO. Similarly, Pandey et al. (2022) reported that silver nanoparticle‐loaded LGEO nanoemulsions exhibited synergistic antimicrobial and antioxidant activity, with complete inhibition of *E. coli* at 0.75% MIC and AFM‐confirmed membrane damage, supporting their role as nanocomposites for active packaging applications. These findings broaden the scope of LGEO nanoemulsions beyond fruit and vegetable preservation.

From an industrial perspective, LGEO nanoemulsions also show promise in liquid and dairy systems. Prakash et al. (2025) reported that vitamin E encapsulated LGEO nanoemulsions (138–183 nm) exhibited strong antibacterial activity against *S. aureus* and *E. coli*, with inhibition zones of 52.5 mm and 23.5 mm, respectively, although stability decreased after 30 days. Nouraddini et al. (2025) showed that soy lecithin‐stabilized LGEO nanoemulsions (0.2%–0.6% w/w) incorporated into orange juice enhanced antioxidant activity (14.38%–77.38%), suppressed bacterial growth during 30 days of refrigeration, and maintained consumer acceptance, confirming their role as natural preservatives in beverages. Salama et al. (2022) further demonstrated that yogurt flavored with LGEO nanoemulsions (< 30 nm) maintains viability of lactic acid bacteria (> 10⁶ CFU/mL), preserves its physicochemical quality, and achieves the highest sensory scores among the tested EOs during 15 days of storage. These studies highlight the versatility of LGEO nanoemulsions as both functional preservatives and flavor‐enhancing agents in complex food matrices.

#### 4.4.2 Microemulsions

Microemulsions have emerged as promising carriers for LGEO in food systems, owing to their ability to form thermodynamically stable, transparent dispersions that enhance solubility, stability, and biological activity of EOs (Bezerra et al. 2023). Recent advances confirm that LGEO microemulsions can deliver broad‐spectrum antimicrobial, antioxidant, and antifungal effects in diverse food matrices. Đekić et al. (2025) demonstrated that film‐forming microemulsions incorporating LGEO and other EOs produced nanosized droplets (13–27 nm) with high thermodynamic stability and optical clarity. These systems enhanced retention and controlled the release of volatile compounds while maintaining antimicrobial and antioxidant properties. Similarly, Alkaabi et al. (2022) demonstrated that aloe vera gel‐based coatings with LGEO microemulsions (1%–5%) significantly inhibited microbial growth, preserved texture, pH, and moisture, and showed strong antifungal activity in soft date fruits. Lee Pei Ling et al. (2019) demonstrated that LGEO blend microemulsions applied in curry paste were transparent, stable across a pH range of 4.1–5.1, and had low viscosity (< 4 mPa s). These microemulsions contained antibacterial compounds that, when combined, inhibited microbial growth, confirming their suitability as natural preservatives for food applications.

Applications in fresh produce preservation highlight LGEO's effectiveness against foodborne pathogens. Arellano et al. (2021) showed that 0.3%–0.5% LGEO microemulsions reduced antibiotic‐resistant *Salmonella* Newport (up to 4.3 log CFU/g) and *Lactobacillus casei* on Iceberg lettuce during 28 days at 4°C. Expanding on this work, Arellano et al. (2022) confirmed that LGEO microemulsions (0.3%–0.5%) decreased *E. coli* O157:H7 populations by 3.7 log CFU/g on lettuce, with no survivors after 3–7 days of storage, while also suppressing *Pseudomonas fluorescens*. Together, these results highlight the potential of LGEO microemulsions as natural sanitizers for minimally processed leafy vegetables.

Beyond antibacterial efficacy, LGEO microemulsions also demonstrate antifungal properties. Suardi et al. (2021) formulated stable microemulsions of LGEO (3%) with clove oil using virgin coconut oil, Tween 80, and PEG 400, achieving inhibition zones of 7.05–8.85 mm against *Trichophyton rubrum*. Nguyen et al. (2024) developed an LGEO‐based microemulsion hydrogel incorporating mango seed extract, achieving droplet sizes of ∼19 nm, sustained release of citral and polyphenols, and strong antibiofilm activity.

#### 4.4.3 Pickering Emulsions

Pickering emulsions are emulsions stabilized by solid particles, rather than conventional surfactants, creating a robust interfacial layer that prevents droplet coalescence and enhances stability (Niroula et al. 2025). This particle‐based stabilization has garnered considerable attention in food applications due to its clean‐label appeal, ability to encapsulate sensitive bioactive compounds, and compatibility with natural polymers such as cellulose, chitosan, and alginate (Fan et al. 2023; Tian et al. 2022; Yu et al. 2023). Encapsulating LGEO in Pickering systems stabilizes it during processing and storage while modulating its release, thereby enhancing efficacy and reducing sensory impacts on foods (Nkede et al. 2023).

Hamzah and Romulo (2025) demonstrated that cellulose nanocrystals and sodium alginate could effectively stabilize LGEO in Pickering emulsions, achieving antimicrobial activity with MIC_50_ values ranging from 4096 to 8192 µg/mL against both Gram‐positive and Gram‐negative bacteria. Beyond laboratory antimicrobial assays, Nkede et al. (2023) provided practical evidence in tomato preservation, where chitosan/lemongrass oil/cellulose nanofiber pickering emulsions reduced weight loss to 9.05% after 15 days, maintained fruit color, and suppressed *Botrytis cinerea* infection by up to 57.44%. Similarly, Wardana et al. (2023) demonstrated that alginate/lemongrass oil/cellulose nanofiber Pickering emulsions inhibited *Penicillium digitatum* and *P. italicum*, achieving up to 91.9% spore germination inhibition while also enhancing coating hydrophobicity and UV–visible light barrier properties.

Studies on the bioactive constituent of LGEO further enrich mechanistic insights. Li et al. (2024) showed that chitosan–sodium alginate/Ca^2^⁺ nanoparticle‐stabilized Pickering emulsions enhanced the thermal, photostability, antioxidant, and antibacterial activities of D‐limonene. Similarly, Wang et al. (2022) reported that citral‐loaded Pickering emulsions exhibited two‐ to eightfold stronger antibiofilm activity than free citral, successfully inhibiting and eradicating biofilms of *S. aureus* and *P. aeruginosa*. In addition, Liu et al. (2022) fabricated chitosan–lignin composite films containing Pickering emulsions of a citral‐rich oil, observing multifunctional improvements, including antimicrobial and antioxidant activities, enhanced barrier performance, and improved mechanical strength.

### 4.5 Micro‐ and Nanoencapsulation

Recent advances show that micro‐ and nanoencapsulation of LGEO with food‐grade materials such as maltodextrin, gum arabic, chitosan, and cyclodextrins improve its thermal and oxidative stability, prolong antimicrobial and antioxidant activity during processing and storage, and enable controlled release in edible films and coatings, thereby extending the shelf life of fruits, vegetables, and dairy products, with increasing evidence of industrial applicability (Moreira da Silva et al. 2024).

Several studies have confirmed the efficacy of microencapsulation in enhancing the stability and functionality of LGEO across various food systems. Martins et al. (2021) reported that the microencapsulation of LGEO in maltodextrin–gelatin matrices, achieved through freeze‐drying, enhanced its thermal and oxidative stability, reduced volatilization, and maintained its antioxidant capacity. Melo et al. (2020) demonstrated that the microencapsulation of LGEO with arabic gum and maltodextrin (1:5, m/m) by spray drying improved thermal stability and oil retention. When applied to Coalho cheese at 0.25%, this treatment effectively inhibited coliform growth at 45°C and extended the shelf life to 21 days. Alencar et al. (2022) further reported that LGEO microencapsulated by spray drying with maltodextrin–gelatin walls (9:1) achieved encapsulation efficiency up to 61.95%, retained total phenolics (1632–4171 µg TE/g), exhibited antioxidant activity (28.55–45.12 µg/g), and antibacterial effects against *S. aureus* and *E. coli*. Magri et al. (2025) showed that encapsulating LGEO in γ‐cyclodextrin at a 3:1 ratio achieved high encapsulation efficiency (78%) and loading capacity (57%), with strong antioxidant activity, up to 92% inhibition of *Botrytis cinerea*, and significant suppression of *Penicillium* spp. In contrast, Rampelotto et al. (2021) demonstrated that dietary supplementation of silver catfish with microencapsulated LGEO (1–3 mL/kg) maintained proximate composition and sensory quality of fillets during 12 months of frozen storage, with no detectable citral residues, while lower doses minimized lipid oxidation and higher doses slightly increased protein oxidation. Collectively, these studies highlight the scalability and versatility of microencapsulation for various food applications.

At the nanoscale, encapsulation strategies provide greater bioactivity, controlled release, and multifunctional applications. Gan et al. (2024) encapsulated LGEO in bilayer liposomes stabilized with chitosan, pectin, gum arabic, or carrageenan and applied them to chicken meat preservation. The biopolymer‐modified nanoliposomes improved storage stability, enhanced antioxidant activity, and delayed spoilage by lowering total volatile base nitrogen and bacterial counts, effectively extending shelf life by up to 12 days. Antonioli et al. (2020) reported that poly(lactic acid) nanocapsules loaded with LGEO (96.4 nm, 99% encapsulation efficiency) inhibited *Colletotrichum acutatum* and *C. gloeosporioides* *in vitro*, and reduced bitter rot lesions in apples by threefold compared to free LGEO. Kumar et al. (2022) confirmed that chitosan–cinnamic acid nanogels encapsulating LGEO (∼147 nm, 41%–77% efficiency) significantly inhibited foodborne molds, suppressed aflatoxin B1 production at 0.4 µL/mL, and protected pearl millet seeds from *A. flavus* contamination and lipid peroxidation during 6 months of storage, while Almeida et al. (2019) optimized LGEO‐loaded PLGA nanoparticles by Box–Behnken design (mean size ∼277 nm, 73% encapsulation efficiency) improved thermal stability, enabled biphasic sustained release of citral for up to 8 days, and reduced cytotoxicity compared to free oil. Soltanzadeh et al. (2021) showed that chitosan nanoparticles encapsulating LGEO (175–235 nm) improved its thermal stability (from 126°C to 252°C), provided pH‐dependent controlled release, and enhanced antibacterial and antifungal activity against foodborne bacteria and molds. In another study, Sattary et al. (2020) demonstrated that LGEO encapsulated in mesoporous silica nanoparticles (EE 84.2%, ∼50–70 nm) exhibited approximately threefold higher antifungal activity against *Gaeumannomyces graminis* compared to free oil, reducing MIC from 96.8 to 31.8 mg/L, and *in vivo* seed treatments achieved 74.4% disease suppression‐ enhanced further to 84% when combined with alginate coating. More recently, Cruz et al. (2023) used electrospun cassava starch fibers encapsulated with up to 40% LGEO (460–577 nm) exhibited high thermal stability, antioxidant activity (36.7% DPPH, 46.6% OH, 37.2% NO), and strong in situ antifungal efficacy against *Penicillium crustosum* and *A. flavus* in bread, where fibers with 40% LGEO reduced fungal counts during 10 days of storage, while Ashaq et al. (2025) incorporated ultrasound‐assisted nanoencapsulated LGEO into gum arabic and maltodextrin, achieving high encapsulation efficiency (86.5%) and improved thermal stability. When applied further in sodium alginate coatings, the system extended strawberry shelf life by up to 20 days, reducing weight loss, decay, and firmness loss. Li et al. (2022a) introduced an innovative 3D‐printed multifunctional preservation card combining β‐cyclodextrin‐encapsulated lemongrass essential oil (LGEO/β‐CD) with popping candy as a CO_2_‐releasing agent for strawberry storage. The system provided sustained LGEO release, inhibited the growth of *Aspergillus niger* and *Botrytis cinerea*, reduced weight loss and respiration, and extended strawberry shelf life to 8 days at 25°C.

### 4.6 Ingredient for Functional Foods

The growing consumer interest in foods that support metabolic balance, immune defense, and chronic‐disease prevention has directed attention toward plant‐derived bioactives with multifunctional roles. LGEO exemplifies such an ingredient offering not only preservative and sensory benefits but also physiological functionality relevant to metabolic health. Evidence from *in vitro*, *in vivo*, and food‐system studies suggests that LGEO and its components can modulate glucose metabolism, inflammatory signaling, and oxidative stress pathways while contributing to cardiovascular and cellular homeostasis (Widiputri et al. 2018; Kusuma et al. 2024; Mukarram et al. 2022). Within food systems, LGEO exhibits promising bioactivity while retaining functional and sensory compatibility. When incorporated into dairy matrices such as yogurt and ice cream, it preserved α‐glucosidase inhibitory activity (IC_50_ ≈ 14.46 mg/mL) under thermal processing and maintained good consumer acceptance (Santoso et al. 2018). This stability suggests that LGEO can be integrated into functional beverages and fermented foods designed to moderate postprandial glucose levels. Its terpenoid constituents, including citral and geraniol, act as inhibitors of α‐amylase and α‐glucosidase, thus delaying carbohydrate digestion and attenuating glycemic spikes (Naz et al. 2024; Widiputri et al. 2018). *in vivo* studies further support these findings, supplementation of LGEO‐infused tea in diabetic rats reduced fasting blood glucose by up to 60%, improved oral glucose tolerance, and restored pancreatic β‐cell function (Garba et al. 2020). These outcomes were attributed to activation of the Nrf2 antioxidant pathway and downregulation of proapoptotic markers, reflecting LGEO's capacity to mitigate oxidative stress and enhance insulin signaling (Elekofehinti et al. 2020). Collectively, these studies support LGEO's integration into foods aimed at glycemic control.

Beyond glucose regulation, LGEO exhibits strong anti‐inflammatory potential, a key attribute for preventing diet‐related chronic diseases. Its phenolic and terpenoid fractions inhibit cyclooxygenase (COX‐2) and lipoxygenase (LOX) enzymes, suppress nitric oxide synthesis, and reduce inflammatory cytokine production (Kusuma et al. 2024). LGEO serves as a bioactive modulator capable of mitigating oxidative stress and inflammation through the reduction of ROS and NO production (Almuhayawi et al. 2021). Recent studies have explored the incorporation of LGEO into beverages and dairy systems, where it demonstrates excellent stability during processing and storage, thereby enabling its application in functional foods aimed at reducing inflammation (Agnish et al. 2022). *in vivo* studies have confirmed that topical or oral administration of LGEO attenuates edema, erythema, and leukocyte infiltration in inflamed tissues (Solon et al. 2025).

The same biochemical mechanisms underlying LGEO's antioxidant and anti‐inflammatory effects also contribute to its emerging role in cellular protection and anticancer activity. Citral, geraniol, and α‐bisabolol have been shown to induce apoptosis, arrest cell cycle progression, and inhibit angiogenesis across multiple cancer cell lines, including breast, cervical, colorectal, and melanoma models (Balusamy et al. 2020; Maruoka et al. 2018). Citral selectively suppresses proliferation in breast cancer cells (MCF‐7, MDA‐MB‐231) without affecting normal epithelial cells, Geraniol further exhibits antiproliferative effects on intestinal adenocarcinoma (Caco‐2) cells through polyamine metabolism disruption (Crespo et al. 2020), an observation particularly relevant for dietary exposure, since Caco‐2 cells are widely used as a model for intestinal absorption in food system. Importantly, delivery systems not only stabilize LGEO's volatile compounds but also enhance their bioactivity. Selim et al. (2025) demonstrated that nanoencapsulated LGEO exhibited stronger cytotoxicity against tumor cells than free oil, alongside antioxidant and antimicrobial properties.

## 5 Safety, Toxicology, and Regulatory for Food Use

The safety and toxicological evaluation of LGEO is central to its acceptance as a natural preservative and flavoring agent in the food industry. Evidence from *in vivo* animal studies consistently shows that LGEO has a low toxicity profile. Acute oral toxicity tests in mice have reported an LD_50_ of approximately 3500 mg/kg, indicating relatively low acute toxicity. Repeated‐dose studies over 21 days, with doses ranging from 1 to 100 mg/kg, revealed no significant effects on body weight, organ weights, hematology, serum biochemistry, or histopathology of major organs, and no genotoxicity was detected (Costa et al. 2011). At higher doses, up to 2000 mg/kg body weight/day for 21 days, no mortality, clinical signs of toxicity, or adverse organ pathology were observed, confirming a wide margin of safety (Rosol et al. 2023). In addition, acute oral toxicity tests at 2000 mg/kg reported no observable toxic symptoms (Chanotiya et al. 2024). To date, however, direct *in vivo* toxicity studies in humans have not been reported, and further research is needed to confirm safety in human populations.

The favorable toxicological profile of LGEO is complemented by its well‐documented antimicrobial and antioxidant properties, further enhancing its value as a functional ingredient for food preservation. The presence of bioactive compounds such as citral, citronellal, geraniol, and myrcene contributes not only to its characteristic aroma but also to its ability to inhibit bacterial and fungal growth, suppress foodborne pathogens, and delay oxidative spoilage (as discussed in Section 2). These activities reduce reliance on synthetic preservatives, thereby enhancing the natural and “clean‐label” appeal of food products. However, despite its benefits, the concentration of LGEO used in foods must be carefully managed. At higher levels, LGEO may impart strong citrus‐like flavors that can dominate or alter the sensory profile of foods. This sensory limitation can be mitigated by combining LGEO with other plant extracts or EOs to achieve synergistic antimicrobial effects while keeping concentrations below the threshold at which flavor modifications become unacceptable (Bag et al. 2022).

From a regulatory perspective, LGEO has a well‐established approval status. The FDA has classified LGEO as “GRAS”, confirming its suitability for food use under prescribed conditions (Bajaj et al. 2024; Magri et al. 2025). This approval has facilitated its widespread application across food categories. However, specific regulatory‐approved concentrations for food use are not available, and their application should be governed by good manufacturing practices (GMP) to avoid exceeding acceptable sensory limits. It is used in alcoholic and nonalcoholic beverages, frozen dairy desserts, candies, baked goods, gelatins, puddings, and minimally processed fruit products (Skaria et al. 2006). In baked goods, such as bread, LGEO serves both as a flavoring agent and as an antifungal/antioxidant additive, with applications extending to active packaging systems (Cruz et al. 2023). Meat, fish, chocolates, and oils also incorporate LGEO for its combined sensory and preservative benefits, and its incorporation into juices and fresh produce demonstrates its potential in extending the shelf life of perishable items (Salvia‐Trujillo et al. 2014). The versatility of these applications highlights LGEO's dual role in enhancing flavor and ensuring microbial safety.

Another important consideration is LGEO's environmental and consumer safety in food systems. Unlike many synthetic preservatives, LGEO is biodegradable and has a low environmental impact, making it a sustainable option for the food industry. While it is generally expected that its volatile, naturally occurring compounds would not persist in the environment or accumulate in food chains, direct experimental confirmation of this remains limited, highlighting the need for further research. Nevertheless, its natural origin and biodegradability align with modern regulatory and consumer expectations, which increasingly emphasize sustainability, reduced ecological footprint, and the safety of food additives.

## 6 Challenges and Prospects

Despite its wide‐ranging biological and food‐related applications, the broader industrial utilization of LGEO faces several critical challenges. One of the main issues among these is its inherent volatility and thermal instability (Erminawati et al. 2019). LGEO's major bioactive components, particularly citral, citronellal, and geraniol, are susceptible to oxidation, heat, and light, which significantly limit their stability in conventional formulations (Ashaq et al. 2024). These characteristics limit LGEO's use in processes that require high temperatures or long‐term storage, particularly in food and therapeutic applications. Another major challenge is its variability in chemical composition. Factors such as geographic origin, harvest season, plant maturity, and extraction technique lead to significant variations in the concentration of active compounds (Skaria et al. 2006). Such inconsistency complicates standardization, affects efficacy, and hinders regulatory approval, particularly in medicinal and therapeutic applications. Moreover, LGEO's hydrophobic nature and strong aroma limit its direct incorporation into aqueous food matrices, where sensory compatibility and consumer acceptance are crucial (Ibáñez et al. 2020).

Stabilization technologies have emerged to address some of these hurdles. Techniques such as microencapsulation and nanoemulsification have shown promise in preserving LGEO's functional integrity and enhancing its shelf life and controlled release. Microencapsulation using maltodextrin, gum arabic, or cyclodextrins has been demonstrated to protect LGEO from environmental degradation while maintaining its antimicrobial and antioxidant activities (Erminawati et al. 2019). Similarly, nanoemulsions enhance the solubility and bioavailability of LGEO, resulting in a more uniform dispersion in food systems and improving microbial inhibition (Barradas and de Holanda e Silva 2020). However, these approaches have limitations, including high processing costs, complex equipment requirements, and a lack of standardized protocols for industrial‐scale applications. Beyond formulation issues, LGEO also presents significant processing challenges that hinder its broader adoption. Conventional techniques such as steam distillation, spray drying, and pasteurization often result in the thermal degradation or evaporation of key volatile constituents, notably citral and geraniol (Mahanta et al. 2021). In food systems, LGEO's poor miscibility in aqueous media can lead to phase separation or uneven distribution, particularly when exposed to varying pH levels, salt concentrations, or thermal treatments during processing (Fathi et al. 2021). These issues are compounded in scaled‐up manufacturing environments, where maintaining LGEO's stability and bioactivity under thermal or mechanical stress is more difficult. Advanced cold‐processing technologies, such as ultrasound‐assisted emulsification, electrospinning, or freeze‐drying‐based encapsulation, may offer better protection for LGEO; however, these are still limited by cost, complexity, and regulatory uncertainty. Addressing these barriers requires a deeper understanding of LGEO's physicochemical behavior during various processing stages, as well as the development of process‐compatible delivery systems that retain functionality without compromising product integrity.

Regulatory and toxicological concerns also remain underexplored. Although LGEO is classified as GRAS, the acceptable use limits across diverse food systems, especially in nanoformulations, have not been thoroughly investigated (Faheem et al. 2022). Studies are needed to evaluate the potential cytotoxicity and allergenicity of bioactive constituents, such as luteolin derivatives, when used in long‐term or high‐dosage applications. Additionally, consumer perception and acceptance of LGEO‐infused products are critical determinants for market success, yet remain vastly underreported. From a production standpoint, the scalability of high‐quality LGEO remains a constraint. Traditional farming and distillation practices often yield oils with inconsistent compositions and low citral content. There is a pressing need for advanced agronomic strategies, including the genetic improvement of *Cymbopogon* species to enhance bioactive yield and citral concentration. Integrating precision agriculture, tissue culture propagation, and optimized postharvest drying techniques can ensure consistent quality and high‐volume production, meeting industrial demands. Looking ahead, integrating LGEO into biodegradable polymers for edible films and active packaging holds substantial promise for reducing the use of synthetic preservatives and minimizing environmental waste. Its incorporation into gelatin, starch, and cellulose‐based matrices has already demonstrated effective antimicrobial and antioxidant properties, crucial for extending the shelf life of perishable food products such as meat, dairy, and produce. Moreover, combining LGEO with other natural preservatives or synergistic phytochemicals could further enhance its functional profile while reducing the concentration required for efficacy (Kiani et al. 2022). Future research should prioritize multiomics approaches to elucidate the molecular mechanisms of LGEO's bioactivity, particularly its interactions at the cellular and genomic levels in pathogenic and probiotic organisms. Furthermore, clinical trials and *in vivo* studies will be crucial for validating LGEO's therapeutic efficacy and safety profile.

## 7 Conclusion

Evidence from numerous studies demonstrates that LGEO is effective across a wide range of food categories, including beverages, dairy, baked products, meat, fish, fruits, and minimally processed produce, where it functions simultaneously as a flavoring and a preservative. Results summarized in this review confirm that LGEO can suppress foodborne pathogens such as *E. coli*, *Salmonella*, *L. monocytogenes*, and *S. aureus*, while also inhibiting spoilage fungi and delaying oxidative deterioration. These outcomes are further enhanced when LGEO is applied through films, coatings, emulsions, or encapsulation systems that improve its stability and control release. Together, these findings highlight LGEO as a versatile and eco‐friendly alternative to synthetic preservatives. However, challenges remain in ensuring compositional consistency across different chemotypes and extraction methods, and in addressing sensory impacts that limit its application at higher concentrations. Strengthening toxicological evidence in humans, alongside optimizing delivery systems and developing standardized formulations, will be crucial for industrial adoption.

## Author Contributions

**Ahmad Rabbani**: writing – original draft, visualization. **Ayman Khaliq**: writing – original draft. **Priti Mudgil**: writing – review and editing. **Sajid Maqsood**: writing – review and editing. **Akmal Nazir**: conceptualization, supervision, writing – review and editing.

## Conflicts of Interest

The authors declare no conflicts of interest.
